# A Combined Transcriptomic and Genomic Analysis Identifies a Gene Signature Associated With the Response to Anti-TNF Therapy in Rheumatoid Arthritis

**DOI:** 10.3389/fimmu.2019.01459

**Published:** 2019-07-02

**Authors:** Adrià Aterido, Juan D. Cañete, Jesús Tornero, Francisco Blanco, Benjamín Fernández-Gutierrez, Carolina Pérez, Mercedes Alperi-López, Alex Olivè, Héctor Corominas, Víctor Martínez-Taboada, Isidoro González, Antonio Fernández-Nebro, Alba Erra, María López-Lasanta, Mireia López Corbeto, Núria Palau, Sara Marsal, Antonio Julià

**Affiliations:** ^1^Rheumatology Research Group, Vall d'Hebron Research Institute, Barcelona, Spain; ^2^Department of Experimental and Health Sciences, Universitat Pompeu Fabra, Barcelona, Spain; ^3^Rheumatology Department, Hospital Clínic de Barcelona and Institut d'Investigacions Biomèdiques August Pi i Sunyer (IDIBAPS), Barcelona, Spain; ^4^Rheumatology Department, Hospital Universitario De Guadalajara, Guadalajara, Spain; ^5^Rheumatology Department, INIBIC-Hospital Universitario A Coruña, A Coruña, Spain; ^6^Rheumatology Department, Hospital Clínico San Carlos, Madrid, Spain; ^7^Rheumatology Department, Parc de Salut Mar, Barcelona, Spain; ^8^Rheumatology Department, Hospital Universitario Central de Asturias, Oviedo, Spain; ^9^Rheumatology Department, Hospital Universitari Germans Trias i Pujol, Barcelona, Spain; ^10^Rheumatology Department, Hospital Moisès Broggi, Barcelona, Spain; ^11^Rheumatology Department, Hospital Universitario Marqués de Valdecilla, Santander, Spain; ^12^Rheumatology Department, Hospital Universitario La Princesa, IIS La Princesa, Madrid, Spain; ^13^UGC Reumatología, Instituto Investigación Biomédica Málaga, Hospital Regional Universitario, Universidad de Málaga, Málaga, Spain; ^14^Rheumatology Department, Hospital Sant Rafael, Barcelona, Spain

**Keywords:** rheumatoid arthritis, genomics, transcriptomics, multi-omics association analysis, anti-TNF therapy

## Abstract

**Background:** Rheumatoid arthritis (RA) is the most frequent autoimmune disease involving the joints. Although anti-TNF therapies have proven effective in the management of RA, approximately one third of patients do not show a significant clinical response. The objective of this study was to identify new genetic variation associated with the clinical response to anti-TNF therapy in RA.

**Methods:** We performed a sequential multi-omic analysis integrating different sources of molecular information. First, we extracted the RNA from synovial biopsies of 11 RA patients starting anti-TNF therapy to identify gene coexpression modules (GCMs) in the RA synovium. Second, we analyzed the transcriptomic association between each GCM and the clinical response to anti-TNF therapy. The clinical response was determined at week 14 using the EULAR criteria. Third, we analyzed the association between the GCMs and anti-TNF response at the genetic level. For this objective, we used genome-wide data from a cohort of 348 anti-TNF treated patients from Spain. The GCMs that were significantly associated with the anti-TNF response were then tested for validation in an independent cohort of 2,706 anti-TNF treated patients. Finally, the functional implication of the validated GCMs was evaluated via pathway and cell type epigenetic enrichment analyses.

**Results:** A total of 149 GCMs were identified in the RA synovium. From these, 13 GCMs were found to be significantly associated with anti-TNF response (*P* < 0.05). At the genetic level, we detected two of the 13 GCMs to be significantly associated with the response to adalimumab (*P* = 0.0015) and infliximab (*P* = 0.021) in the Spain cohort. Using the independent cohort of RA patients, we replicated the association of the GCM associated with the response to adalimumab (*P* = 0.0019). The validated module was found to be significantly enriched for genes involved in the nucleotide metabolism (*P* = 2.41e-5) and epigenetic marks from immune cells, including CD4+ regulatory T cells (*P* = 0.041).

**Conclusions:** These findings show the existence of a drug-specific genetic basis for anti-TNF response, thereby supporting treatment stratification in the search for response biomarkers in RA.

## Introduction

Rheumatoid arthritis (RA) is the most common autoimmune-inflammatory arthritis affecting up to 1% of the worldwide population (1). RA is characterized by the chronic infiltration of immune cells in the synovial membrane, leading to progressive destruction of the joint cartilage and bone (2). The most notable success in the treatment of RA has been the introduction of Tumor Necrosis Factor (TNF) inhibitors. Anti-TNF agents have radically changed the prognosis of many RA patients (3), providing an important improvement of clinical signs and symptoms, quality of life and long-term protection of the synovial joint integrity. Despite this major accomplishment, there is a large fraction of anti-TNF treated patients (30–40%) that do not show a significant clinical improvement (4). To date, little is known on the biological mechanisms that underlie this differential response to anti-TNF agents. Understanding the basis of the lack of response could not only help to personalize patient therapy but also to gain insights into RA heterogeneity at the molecular level.

The two main classes of anti-TNF agents are monoclonal antibodies against TNF, like adalimumab and infliximab (5, 6), and the recombinant fusion protein containing the soluble TNF receptor p75 etanercept (7). Despite targeting the same molecule, the different anti-TNF drugs do not show the same level of efficacy in all patients. At the cellular and molecular level, there is evidence that the clinical response is partially mediated by the activation of drug-specific biological mechanisms (8–10). Supporting this, clinical observations have shown that patients who fail one anti-TNF treatment may still respond to a different anti-TNF drug (11). Therefore, there is a need to identify the genetic variability underlying the response to anti-TNF agents.

To date, a small number of genome-wide transcriptomic studies have been conducted on the RA synovium to investigate the biological processes associated with anti-TNF response (12–17). The gene expression signatures obtained in these studies, however, have shown a modest association with the treatment outcome. As a result, the overlap of differentially expressed genes between these studies is relatively low (18). This evidence suggests the existence of a high biological variability between RA patients. Identifying the source of this biological variation would be a major advance toward personalized medicine in RA (19).

Associating genetic variation to disease risk has provided a wealth of genes and biological pathways relevant for RA (20). The use of this approach to characterize the genetic basis of anti-TNF response in RA has, however, proven less productive. More than 40 candidate-gene association studies have been performed so far, but there has been very limited or non-existent replicability (21). Genome-wide association studies (GWAS) have proven to be a more successful approach for this objective. To date, eight GWAS on anti-TNF response in RA have been performed (22–29), identifying several loci associated at a genome-wide scale. From these, variation at *MED15, GFRA1, PDE3A-SLCO1C1*, and *CD84* has been replicated in, at least, an independent cohort of patients (30). However, these few loci are insufficient to explain the heritability of anti-TNF response and, consequently, alternative analysis approaches must be devised (31, 32).

The integration of high-throughput transcriptomic and genomic data offers a new opportunity to characterize the biological basis underlying complex traits (33–37). In RA, the integrative analysis of gene expression levels and genetic variation has proven effective to identify novel causal genes as well as cell-type specific mechanisms associated with the disease pathogenesis (38–41). As an example, gene expression data from peripheral blood mononuclear cells has been successfully used to guide the selection of candidate genes for genetic association analysis (42). Accordingly, analyzing the expression profile associated with anti-TNF response at the target site of inflammation in RA -the synovial membrane- should be a powerful means to identify genetic variation associated with the therapeutic response. To date, this type of integrative analysis has not been performed in RA.

To gain a better understanding of the biological mechanisms of anti-TNF response in RA, we have performed a combined transcriptomic and genomic analysis. Using transcriptomic data from the RA synovium, we first identified the modules of co-expressed genes that are associated with anti-TNF response. We next tested the association of these modules at the genetic risk level using two independent GWAS cohorts of RA patients. Finally, we investigated the functional implication of the genetic modules associated with anti-TNF response at the two levels of molecular variation. These findings demonstrate the power of integrating multi-omic data to characterize the genetic basis of drug response in a complex disease like RA.

## Materials and Methods

### Study Population

#### RA Cohort of Patients Used for the Genome-Wide Expression Profiling

A total of 11 RA patients with clinically active RA from Spain were recruited by the outpatient's clinics of the rheumatology department of the Hospital Clinic de Barcelona. All patients fulfilled the 1987 American College of Rheumatology (ACR) classification criteria for RA (43). The main epidemiological and clinical variables of these patients are summarized in Table 1.

**Table 1:** Clinical and epidemiological characteristics of the patient cohort used for the genome-wide expression profiling.

**Characteristic**	**RA cohort transcriptomic analysis**
Population	Spain
Disease diagnosis	1987 ACR criteria for RA
Molecular data	RNA
Sample size (*N*)	11
Gender (female, %)	73.01
Naïve to anti-TNF therapy (%)	100.00
Previous therapies (MTX, %; LFD, %)	100.00; 81.81
Responders to anti-TNF therapy (*N*)	5
Moderate responders to anti-TNF therapy (*N*)	3
Non-responders to anti-TNF therapy (*N*)	3

*ACR, American College of Rheumatology; MTX, methotrexate; LFD, leflunomide; N, sample size; RA, rheumatoid arthritis; TNF, tumor necrosis factor*.

#### Discovery Cohort of RA Patients Treated With Anti-TNF Therapy

A total of 348 RA patients that had received an anti-TNF treatment as their first biological treatment (adalimumab, infliximab, or etanercept) were included in the discovery stage of the genetic association study. This cohort of RA patients was collected from the outpatient's clinics of the rheumatology departments of 12 Spanish University Hospitals involved in the Immune-Mediated Inflammatory Disease Consortium (IMIDC) (44). For this study, all patients fulfilled the 1987 ACR classification criteria for RA and had more than 2 years of follow-up since diagnosis (43). All recruited individuals had an erosive disease defined as erosions in more than one joint group including hands and/or feet.

All RA patients included in this cohort were Caucasian European and born in Spain. Only those RA patients with all grandparents born in Spain were selected for the set-based genetic association analysis. The main clinical and epidemiological characteristics of this cohort of RA patients are summarized in Table S1.

#### Replication Cohort of RA Patients Treated With Anti-TNF Therapy

Validation of the associated modules was performed using the RA anti-TNF therapy pharmacogenetic cohort described previously (22). This cohort consists of 2,706 RA patients of European ancestry compiled from 13 collections across five countries that had received an anti-TNF treatment (adalimumab, infliximab, or etanercept). All patients fulfilled the 1987 ACR criteria for RA or were diagnosed by a board-certified rheumatologist as previously described (22). The main clinical and epidemiological characteristics of this independent cohort are summarized in Table S1.

### Ethics Statement

This study was carried out in accordance with the recommendations of the guidelines and regulations of the Hospital Universitari Vall d'Hebron Clinical Research Ethics Committee and local institutional review boards with written informed consent from all subjects. All subjects gave written informed consent in accordance with the Declaration of Helsinki. The protocol was approved by the Hospital Universitari Vall d'Hebron Clinical Research Ethics Committee.

### Clinical Response Definition

The clinical response to anti-TNF therapy was measured using the European League Against Rheumatism (EULAR) treatment response criteria (45). For all patients, the Disease Activity Score (DAS28) was measured at baseline and after 3–6 months of anti-TNF therapy (46). According to the DAS28 variation and the DAS28 at the endpoint, RA patients were categorized into good, moderate, and non-responders (Table S1).

### Synovial Biopsy and RNA Extraction

Synovial biopsies were obtained by guided arthroscopy of the inflamed knee joint from 11 RA patients using a 2.7 mm arthroscope (Storz, Tuttlingen, Germany) under local anesthesia. In all patients, 6–8 biopsies were extracted from the suprapatellar pouch and medial and lateral gutters with a 3 mm grasping forceps. According to previous pharmacogenetic studies demonstrating that the clinical response to anti-TNF therapy in RA is associated with the pre-treatment transcriptomic profile (12), synovial biopsies were obtained before the treatment initiation. Each synovial sample was snap-frozen in liquid nitrogen and stored at −80°C for later RNA extraction. Total RNA was extracted from the synovial biopsies using the RNA Mini Kit (Qiagen, USA) and the integrity was assessed using BioAnalyzer microfluidic gel analysis (Agilent, USA). All samples were of high quality (RNA Integrity Number > 8). After RNA isolation, biotin-labeled cRNA (1.5 μg) was prepared using the Ambion Illumina RNA amplification kit (Ambion, USA) and Illumina TotalPrep RNA Amplification Kit (Ambion, US).

### Genome-Wide Gene Expression Profiling

Whole genome transcript abundance from the RA inflamed synovium of the 11 RA patients was performed using the Illumina HumanWG-6 expression array system (Illumina, San Diego, CA, USA). This microarray platform measures the gene expression levels of more than 47,000 different transcripts. Data collection was performed using BeadStudio 3.1.1.0 software (Illumina, San Diego, USA). In order to use the most recent human genome annotation information, only microarray probes matching curated gene sequences from NCBI RefSeq database (release 51) (47) were selected. A final number of 21,189 curated probes representing the expression of 18,524 different human genes were finally selected for analysis. The gene expression intensities were then normalized on the log2-scale using the quantile normalization method (48). The presence of a potential bias between batches of microarray was minimized using the Bayesian ComBat procedure (49). The Raw and normalized data analyzed in the present study are publicly available in the NCBI's Gene Expression Omnibus database (accession number: GSE47726) (50).

### Genome-Wide Coexpression Analysis

There is compelling evidence that the expression of human genes is highly coordinated to develop biological functions (51). In order to identify modules of genes that might share transcriptional regulatory mechanisms in the RA inflamed synovium, we performed a genome-wide co-expression analysis using transcriptomic data from the synovial biopsies of 11 RA patients. For this objective, we used the weighted correlation network analysis (WGCNA) implemented in R software (52). In this method, the absolute pairwise gene expression correlation is raised to a soft thresholding power (β = 14) to compute a network adjacency matrix that emphasizes high correlations at the expense of low correlations. This matrix determines the gene coexpression network that is subsequently used to identify groups of genes with a high topological overlap (53). In this step, an unsupervised hierarchical clustering approach was implemented to detect the most representative modules of co-expressed genes in the RA inflamed synovium. Those modules composed by 10–300 genes were selected for follow-up analyses (54).

In order to describe how each GCM fits with genetic findings from previous studies on anti-TNF response in RA, we assessed the gene overlap of each GCM in genes that have been associated with anti-TNF response at the transcriptomic (*N* > 500 differentially expressed genes in the RA synovium) (12, 14–16, 18) and genetic level (*N* = 78 genes, *P* < 1e-05 in GWAS catalog) (55). In addition, we have investigated whether the GCMs include susceptibility genes for RA (*N* = 200 genes, *P* < 5e-08 in GWAS catalog) (55).

### Association Analysis Between Gene Coexpression Modules and the Clinical Response to Anti-TNF Therapy at the Transcriptomic Level

In order to analyze the association between each gene coexpression module (GCM) and the clinical response to anti-TNF therapy, we performed a principal component analysis (PCA). The first principal component, which captures the largest variability of the GCM (i.e., GCM eigengene), was used to test for association with anti-TNF response using a logistic regression model adjusted by gender. Based on the hypothesis that more extreme response phenotypes provide improved discrimination, we compared EULAR good responders (*N* = 5 patients) and EULAR non-responders (*N* = 3 patients) as previously described (22). Individuals showing an EULAR moderate response were excluded from the analysis (*N* = 3 patients). The complete list of association results for each GCM are shown in Table S2.

The GCMs that showed a significant association with the clinical response to anti-TNF were further characterized by computing the module significance and intramodular connectivity using the WGCNA software. The module significance is a measure of the biological significance of a given module for the phenotype tested for association. This measure is defined as the absolute value of the average biological significance of each gene and ranges from 0 to 1 (i.e., the higher the module significance, the more biologically significant is the module for the phenotype tested for association). The intramodular connectivity is an average measure of the gene connectivity within a given module.

### Genetic Variation at the Gene Coexpression Modules Associated With Anti-TNF Response

#### GWAS Data From the Discovery Cohort of RA Patients

The association between GCMs and anti-TNF response was also studied at the genetic level. For this objective, we used genotype data from the discovery cohort of RA patients to investigate the impact of genetic variation at these GCMs on the clinical response to anti-TNF therapy (25). The detailed procedure that was followed to perform genome-wide genotyping with the Illumina Quad610 BeadChip (Illumina, San Diego, California, USA) and the quality control analysis have been described previously (25). To evaluate the presence of population stratification in the anti-TNF treated patients, we conducted a PCA implemented in EIGENSOFT (v4.2) software (56). Using the first 10 principal components of variation over 10 iterations, none of the samples showed an outlier genetic background (Figure S1). To increase the number of genetic variants available for association testing, we performed genome-wide imputation. After pre-phasing the haplotypes using SHAPEIT V2 (Oxford, UK), imputation was conducted with IMPUTE V2 (Oxford, UK) (57). As a reference, we used the Caucasian European cohort (*N* = 379 samples) data generated by the 1,000 Genomes Project (phase 1, version 3) (58). Only those SNPs showing a MAF > 0.05 and having a high-quality imputation score (i.e., info quality metric > 0.8) were selected for the set-based genetic association analysis. After imputation, a total of 1,387,382 genetic variants were finally available to be tested for association with the clinical response to anti-TNF therapy.

#### GWAS Data From the Replication Cohort of RA Patients

In order to test for replication of the GCMs that were found to be associated with anti-TNF response in the discovery cohort, we used GWAS data from an independent cohort of 2,706 RA patients that had received anti-TNF treatment. These GWAS data were obtained from the Synapse public repository (syn3280809, doi: 10.7303/syn3280809)). The genotyping and imputation procedures have been previously described (22). A total of 2,539,607 genetic variants were available for replication testing of the GCMs associated in the discovery cohort.

### Set-Based Genetic Association Analysis

The set-based genetic association analysis is a powerful approach to test the association between groups of biologically-related genes and complex traits (59, 60). Using this analytical strategy we have successfully identified new genetic pathways associated with psoriasis, psoriatic arthritis, and with clinically relevant phenotypes of systemic lupus erythematosus (61–63). In the present study, we have used the set-based test implemented in PLINK. In order to use this analytical methodology, SNPs need to be assigned to genes and, subsequently, to GCMs. For this objective, we conducted the SNP-gene mapping using proximity-based criteria, which is the pre-dominant approach in genetic association analyses at the set level (54, 59). According to the reference studies using this statistical approach (59, 64, 65), we used a SNP-gene distance window of 20 Kb and the NCBI RefSeq database for gene annotation (Release 63, 12th October 2017) (66). Those SNPs mapping to genes that are included in GCMs (Table S2) were subsequently assigned to their corresponding GCMs. A total of 249,220 SNPs mapping to the transcriptomically-associated GCMs were finally available for set-based analysis at the genetic level. In the set based-testing, an average association statistic is computed for each GCM and its empirical significance is calculated by a permutation-based approach. Briefly, SNPs are pruned for LD and then each one is individually tested for association with the phenotype, in this case, response to anti-TNF therapy. Association testing was here performed using a logistic regression model incorporating the principal components of variation to control for potential ancestry variation. In the same way of the transcriptomic analysis, genetic association of each GCM was performed using only patients with more distinct responses: that is, EULAR good responders and EULAR non-responders. The *P*-values of the associated GCMs in the discovery and validation cohorts were combined using the logit method implemented in R (67).

### Functional Characterization of the Adalimumab-Associated Gene Coexpression Module

#### Enrichment Analysis in Common Biological Pathways

In order to investigate the biological relevance of the GCM associated with the clinical response to adalimumab, we conducted a functional enrichment analysis on common biological processes. For this objective, we used the reference databases for biological pathway annotation: (i) BioCarta (www.biocarta.com), (ii) Kyoto Encyclopedia of Genes and Genomes (KEGG) (68), and Reactome (69). To ensure that the enrichment results are representative of the biology underlying the adalimumab response, we used exclusively those genes that were associated with treatment response in both the discovery and replication cohorts (*P* < 0.05). The enrichment analysis was performed using the hypergeometric statistical test (70) and the False Discovery Rate (FDR) method was used to account for multiple testing (71).

#### Cell Type Epigenetic Enrichment Analysis

Many of the pathological cell types responsible of the clinical heterogeneity of autoimmune diseases are still unknown (72). In the present study, we hypothesized that genetic variation from the associated modules is a valuable source of biological information to identify cell types influencing the anti-TNF response. Accordingly, we analyzed the enrichment of the module-associated variation on the cell-type-specific H3K4me3 chromatin mark using the EPIGWAS software (73). For this analysis we used the module-associated variation (*P* < 0.05 in either the discovery or replication cohorts), epigenetic data from H3K4me3 peaks of 34 cell types generated by the Roadmap project (74), and reference genotyping data from the Caucasian European population included in the 1,000 Genomes Project (58). The EPIGWAS software estimates the regulatory score of each variant (i.e., normalized ratio between the nearest H3K4me3 peak and distance to the summit of the peak). For each cell type, the loci scores are summed to assess the cell type regulatory score. Using a sampling-based approach (*N* = 10,000 matched sets of SNPs from the HapMap Project), the statistical significance of the enrichment analysis is defined as the proportion of matched sets exceeding the observed cell type specific regulatory score. In addition to this cell type epigenetic enrichment analysis, we have also conducted an epigenetic fine-mapping of the module-associated variation in enhancer histone marks previously described as cell-type specific (i.e., H3K27ac and H3K4me1) (75). This analysis has been performed using HaploR software (76, 77).

## Results

### RA Synovium Gene Coexpression Modules Associated With Anti-TNF Response

**Figure 1:**
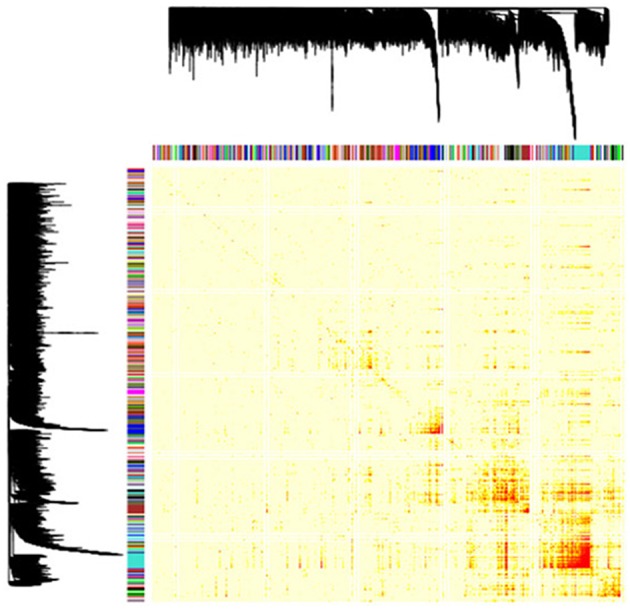
Gene coexpression modules identified in the RA inflamed synovium. Dendrogram showing the 149 gene coexpression modules identified with the unsupervised hierarchical clustering approach in the genome-wide coexpression analysis. Each dendrogram branch corresponds to a gene coexpression module, as shown in the color bar below. The height of the dendrogram represents the co-expression distance among genes. The heatmap represents the adjacency matrix that was built using the pairwise gene expression correlation raised to a soft thresholding power of β = 14. The heatmap is colored according to the absolute value of the pairwise gene expression correlation, ranging from yellow (i.e., weak correlation) to red (i.e., strong correlation). The *x* and *y* axis represent the total of 18,524 genes that were included in the genome-wide coexpression analysis.

To determine the expression patterns that characterize the inflamed synovium in RA, we performed a genome-wide weighted coexpression analysis on transcriptomic data generated from synovial biopsies from patients starting anti-TNF therapy. Using this approach, we identified a total of 149 GCMs (Figure 1). The module content ranged from 14 to 251 genes (Table S2). From the total of 149 GCMs identified in the genome-wide coexpression analysis, we found that 74 GCMs (49.66%) include genes that are differentially expressed between responders and non-responders to anti-TNF therapy, 9 GCMs (6.05%) include genetic variation underlying anti-TNF response, and 37 GCMs (24.83%) include susceptibility genes for RA (Table S3).

We then analyzed the association between the 149 GCMs and the response to anti-TNF therapy at week 14. We found that 13 GCMs were significantly associated to anti-TNF response (*P* < 0.05, Table 2).

**Table 2:** Gene coexpression modules from the RA inflamed synovium that are associated with anti-TNF response at the transcriptomic level.

**Gene coexpression module^a^**	**Gene**	**Module significance**	**Intramodular connectivity (m ± sd)**	***P*-value**
GCM-1	21	0.91	4.03 ± 1.27	0.00021
GCM-2	61	0.81	8.73 ± 2.84	0.0048
GCM-3	52	0.87	7.45 ± 3.05	0.0076
GCM-4	22	0.86	4.6 ± 1.39	0.0077
GCM-5	143	0.86	10.99 ± 4.68	0.014
GCM-6	27	0.66	3.79 ± 1.66	0.019
GCM-7	18	0.68	3.17 ± 0.91	0.028
GCM-8	119	0.77	10.11 ± 4.16	0.033
GCM-9	18	0.79	3.55 ± 0.95	0.033
GCM-10	87	0.78	9.43 ± 3.97	0.036
GCM-11	32	0.78	3.76 ± 1.4	0.037
GCM-12	70	0.74	7.22 ± 2.63	0.038
GCM-13	24	0.69	3.24 ± 1.01	0.046

GCM, gene coexpression module; IMC, intramodular connectivity; M, mean; MS, module significance; SD, standard deviation.

a Gene coexpression modules showing a significant association (P < 0.05) with the anti-TNF response when comparing EULAR good responders (N = 5 biopsies from the RNA inflamed tissues) and EULAR non-responders (N = 3 biopsies from the RNA inflamed tissues) at the transcriptomic level.

### Association of RA Gene Coexpression Modules and Anti-TNF Response at the Genetic Level

After identifying the genetic modules associated with anti-TNF response at the transcript level, we sought to test their association at the genetic level. Using a set-based analysis approach on GWAS data from 348 anti-TNF treated RA patients from Spain, we tested the association of the 13 GCMs with the response to anti-TNF therapy (Table S4). GCM-7 and GCM-10 were found to be significantly associated with the response to adalimumab and infliximab treatments, respectively (*P* < 0.05, Table 3). Using GWAS data from a large international cohort of 2,706 RA patients, we validated the association between GCM-7 and the response to adalimumab (*P* = 0.0019, Table 3).

**Table 3:** Gene coexpression modules showing treatment specific associations at the genetic level.

**GCM characteristics^a^**	GCM-7^*^	**GCM-10**
Module genes	18	87
Anti-TNF agent	Adalimumab	Infliximab
Responders_Disc_ (*N*)	41	30
Non-Responders_Disc_ (*N*)	20	41
Responders_Repl_ (*N*)	438	289
Non-Responders_Repl_ (*N*)	171	244
*P*_Disc_	0.015	0.021
SNPs_Disc_ (*N*)	5,321	1,473
SNPs_DiscSig_ (*N*)	346	209
SNPs_DiscSigLD_ (*N*)	18	18
SNPs_Disc_	rs72681642, rs3104464, rs12135530, rs10782647, rs4562674, rs11165922, chr5:67561207, chr8:12854804, rs2454329, rs62187579, rs140900228, rs11165917, rs1519149, rs10061960, rs1550805, rs9677007, rs4949803, rs74105112	rs934450, rs515247, rs1553544, rs2038442, rs11709521, rs7735205, rs55747890, rs4693803, rs6885000, rs632639, rs62234580, rs2341467, rs8074195, rs11097130, rs6777047, rs1148944, rs11117909, rs3803328, chr7:12288371, chr13:96078081
*P*_Repl_	0.0019	0.567
SNPs_Repl_ (*N*)	15,270	3,760
SNPs_ReplSig_ (*N*)	573	135
SNPs_ReplSigLD_ (*N*)	20	20
SNPs_Repl_	rs2297595, rs6593637, rs12047928, rs1709409, rs17100570, rs6685859, rs10747488, rs7414210, rs12037848, rs2466270, rs11102704, rs7169644, rs11165830, rs17379082, rs17784113, rs6863799, rs12567557, rs1992771	rs16924328, rs6473297, rs4880181, rs7630022, rs3795039, rs1114518, rs2075814, rs2065394, rs259256, rs888246, rs10918341, rs867616, rs10407836, rs17024986, rs6766989, rs2380208, rs13159715, rs2072512, rs7853333, rs11977828
*P*_Comb_	3.02e-4	–

Anti-TNF, anti-tumor necrosis factor treatment associated with the indicated gene coexpression module; Comb, combined p-value (i.e., p-value was only combined for the replicated associations); Disc, discovery cohort; GCM, gene coexpression module; P, empirical P-value obtained in the set-based genetic association analysis; N, sample size; Repl, replication cohort; Sig, nominally associated SNPs; LD, SNPs that are not in linkage disequilibrium (r^2^ < 0.02); SNP, single nucleotide polymorphism.

a Characteristics of the gene coexpression modules that were found to be associated with the indicated treatment in the discovery cohort.

* Replicated associations (P < 0.05 in both the discovery and replication cohorts).

### Adalimumab-Associated Module Highlights the Implication of the Nucleotide Metabolism and Immune Cells on Anti-TNF Response

**Figure 2:**
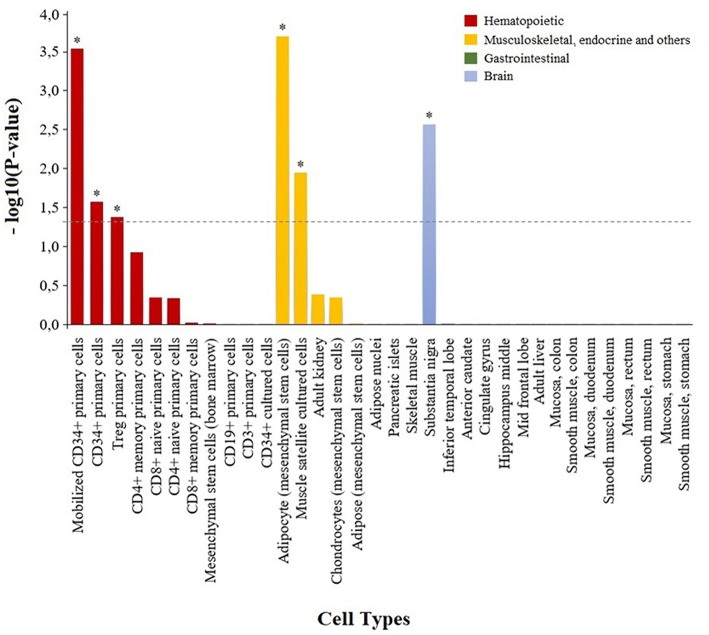
Enrichment of genetic variation from the adalimumab-associated module in H3K4me3 histone marks in specific cell types. Bar plot showing the statistical significance of the adalimumab-associated genetic module and H3K4me3 histone marks from the 34 cell types analyzed. Cell types with H3K4me3 histone marks significantly enriched in adalimumab-associated variants are represented with an asterisk (*P* < 0.05).

To characterize the functional role of the module associated to adalimumab response, we performed two complementary enrichment analyses. Using reference data from common biological pathways, we found that GCM-7 is significantly enriched in genes that participate in the nucleotide metabolism (*P* = 2.41e-05, Table 4; Table S5; Figure S2). Using cell-type specific H3K4me3 epigenetic data, we found that genetic variation at GCM-7 is significantly enriched in epigenetic marks from six different cell types (Figure 2), including CD4+ regulatory T cells (Tregs, *P* = 0.041) and CD34+ myeloid lineage precursors (*P* = 0.021). No significant differences between the number of module-associated variants mapping to the enhancer marks H3K27ac and H3K4me1 were detected in any cell type (*P* > 0.05). Enhancer histone marks from fibroblast primary cells were found to include the largest number of module-associated variants (Figure S3).

**Table 4:** Common biological processes enriched in genes from the adalimumab-associated module.

**Biological pathway^a^**	**Genes^b^**	***P*-value**	**FDR**
Nucleotide metabolism^*^	70	2.41e-05	0.026
GP1b-IX-V activation signaling	10	0.0061	0.068
Interleukin-7 signaling	11	0.0071	0.068
Pyrimidine catabolism	12	0.0071	0.068
Regulation of signaling by CBL	18	0.011	0.068
Synthesis and interconversion of nucleotide di- and triphosphates	18	0.011	0.068
Tie2 signaling	18	0.011	0.068
Signaling by constitutively active EGFR	19	0.011	0.068
CD28 dependent PI3K/Akt signaling	21	0.013	0.068
Nephrin interactions	22	0.013	0.068
Pyrimidine metabolism	24	0.014	0.068
Interleukin receptor SHC signaling	28	0.017	0.072
CD28 co-stimulation	31	0.019	0.072
GPVI-mediated activation cascade	33	0.021	0.072
PI3K/AKT activation	37	0.022	0.072
PI3K events in ERBB4 signaling	38	0.023	0.072
GAB1 signalosome	39	0.023	0.072
Interleukin-2 signaling	42	0.025	0.072
PI3K events in ERBB2 signaling	44	0.026	0.072
Interleukin-3, 5 and GM-CSF signaling	46	0.028	0.072
Downstream TCR signaling	50	0.031	0.072
Mitochondrial protein import	52	0.031	0.072
Antigen activates BCR leading to second messengers' generation	53	0.032	0.072
PI-3K cascade	57	0.034	0.074
TCR signaling	67	0.041	0.081
PI3K cascade	70	0.042	0.081
Costimulation by the CD28 family	76	0.045	0.081
G alpha (12/13) signaling events	77	0.046	0.081
Signaling by SCF-KIT	79	0.047	0.081
IRS-mediated signaling	81	0.048	0.081
IRS-related events	81	0.048	0.081

FDR, false discovery rate.

a Biological processes significantly enriched in the adalimumab-associated module (P < 0.05).

b Number of genes included in the indicated biological process. The complete list of genes can be found in Table S5.

* Biological processes showing a significant enrichment after multiple testing correction (FDR < 0.05).

## Discussion

One of the major challenges in the treatment of RA is to understand the biological mechanisms influencing the clinical response to anti-TNF therapy. Genetic and transcriptomic analyses have been used separately to characterize the molecular basis of treatment efficacy, but with limited success. To investigate the genetic basis of anti-TNF response in RA, we have performed a combined transcriptomic and genomic analysis. Analyzing gene expression data from the RA inflamed synovium, we have first identified the GCMs that are associated with anti-TNF response. We have then tested the association of these GCMs at the genetic level using two independent patient cohorts. This combined genomic approach has enabled to identify a genetic module that is associated with the response to adalimumab. Functional analysis of this module suggests that nucleotide metabolism and Tregs could mediate this response.

In this study, we have found a genetic basis for the clinical response to adalimumab that is not shared with other anti-TNF drugs. These results are in line with previous genetic findings derived from both GWAS and candidate-gene analyses. Genome-wide significant loci *MED15* and *CD84* were found to be associated with the clinical response to etanercept, but not to adalimumab or infliximab (22, 25). At the candidate-gene level, we have previously found that variation at *FCGR2A* gene, which here mapped to a GCM composed by >300 genes that was excluded from the analysis, is associated with the clinical response to adalimumab but not to etanercept (78). There is also evidence of treatment-specific variation at the transcriptomic level. Treatment with adalimumab has been associated with a gene signature in the synovial membrane that is involved in cellular proliferation (16). This gene signature, however, has not been observed after infliximab treatment, thereby suggesting a different mode of action in apparently similar drugs (12). Taken together, our results provide additional support to the existence of specific biological mechanisms that mediate the response to adalimumab.

The genetic module associated with adalimumab response was found to be enriched in genes that participate in the nucleotide metabolism. In addition to the essential role that this biological process plays in DNA replication, nucleotide metabolism is responsible for the synthesis of adenosine, a purine nucleoside that exhibits a potent anti-inflammatory activity when bound to its cognate adenosine receptors (79). Binding of adenosine to the A_2A_ receptor in M1 macrophages, the principal producers of TNF in the synovial joint in RA, induces the switch to the anti-inflammatory M2 phenotype and subsequent reduction of pro-inflammatory mediators (80–82). In turn, adenosine receptors are upregulated by cytokines that activate NFκB, like TNF, and their expression has been found to be high in RA patients (83). Despite this overexpression, adenosine receptors display a weaker affinity for adenosine in RA patients compared to controls, thereby dampening their anti-inflammatory effect. There is evidence that treatment with adalimumab and not methotrexate restores the binding parameters of adenosine receptors of RA patients to those of healthy individuals (84, 85). Our results are in line with this evidence and provide a functional link between the effectivity of an anti-TNF therapy and the local production of adenosine in the synovial joint.

The integration of cell type epigenetic data with our genetic association results suggested an important role of immune cells for mediating the response to adalimumab in RA. In this analysis, both Tregs and CD34+ cells are associated with anti-TNF response. Tregs are central anti-inflammatory and self-tolerance mediators, producing high levels of anti-inflammatory cytokines TGF-β and IL-10 to inhibit the overactivation of effector T cells (86–88). In RA, Tregs have been found to be functionally defective, and treatment with anti-TNF agents has shown to restore their T cell suppressor capacity (89, 90). There is evidence, however, that this beneficial effect on Tregs is reached via treatment-specific mechanisms (91). In particular, adalimumab, but not etanercept, has been shown to induce a particular Treg phenotype that restrains IL-17-mediated inflammation by downregulating the production of IL-6 by monocytes (92). In line with the specific effects that anti-TNF agents can have on different T cell subsets (93), our findings indicate that genetic variation could influence the activity of Tregs of RA patients treated with adalimumab.

In this study, we have identified a GCM that is enriched in epigenetic marks of CD34+ myeloid precursor cells and associated to adalimumab response. There is evidence that circulating bone marrow-derived stem cells like CD34+ cells migrate to the inflamed RA synovium (94), where they form *de novo* blood vessels during the acute phase of the disease (95, 96). Neoangiogenesis is an essential mechanism for the recruitment of immune cells into the RA synovium and perpetuation of the synovial inflammation (97). After recruitment, the high immune cell proliferation generates and hypoxic environment that stimulates the production of more neoangiogenic mediators (79), which have been found highly expressed in the RA inflamed synovium (98–100). From a pharmacological perspective, anti-TNF agents have been shown to ameliorate inflammation by effectively reducing RA synovium vascularity (101, 102). As expected, this reduction has been found to be stronger in responder patients (103). Based on this evidence, the modulation of the neoangiogenic activity of CD34+ cells could be one of the possible mechanisms by which anti-TNF agents could ameliorate synovial inflammation in RA. Supporting this hypothesis, recent studies have identified myeloid signatures and phenotypes in RA synovium that are associated to treatment response (17, 104). Consequently, genetic variation regulating the neoangiogenic function of the CD34+ cells infiltrating the RA synovium could also influence the clinical response to adalimumab.

The present study has limitations. The number of RA patients included in the transcriptomic analysis was relatively low. For this reason, patients could not be stratified by neither anti-TNF agent nor synovial phenotype. It is likely that, by analyzing a larger number of synovial biopsies, additional GCMs and biological mechanisms underlying anti-TNF response in the RA synovium could be identified. Although extracting synovial biopsies from larger cohorts of patients is clinically challenging, this will help to expand the present genetic association results. Another limitation is that the clinical response for the two GWAS cohorts was not identical. While both patient cohorts used the same clinical score (i.e., EULAR response criteria), the Spain sample recorded the response at 3 months of treatment, and the replication sample included clinical responses in a 3–6 months window. The clinical efficacy at these two time points tends to be correlated, however, this difference could have led to a loss of statistical power to validate the genetic module associated with the response to infliximab (GCM-10). Finally, one caveat of the set-based method used here is that the genetic association between GCMs and anti-TNF response was tested using SNPs within or proximal to the GCMs' genes. It is well-known that many regulatory SNPs lie in the non-coding genome and modulate gene expression through 3D interactions and eQTL mechanisms. Our strategy did not account for this. To our knowledge, there is yet no set-based method that has successfully integrated this regulatory information. One of the major problems for this approach is the context-dependent nature of many 3D interactions and eQTLs. In particular, there is evidence that both spatial DNA organization and eQTLs are cell-type and cell-state dependent (105–111). The integration of this regulatory information is therefore still a challenge for the set-based genetic analysis. With the increasing regulatory information that is currently being derived from eQTL analysis and Hi-C experiments on separate cell types, a more profound ascertainment of the impact of SNPs on gene expression will be obtained and, eventually, more comprehensive set-based methods will be developed. These new findings, however, will exponentiate the number of possible regulatory sites, and careful selection of disease-relevant cell types should be performed. To this regard, our study suggests that regulatory T cells and CD34+ progenitors are relevant cell types for the mediation of the response to anti-TNF agents. It is nonetheless important to note that the lack of publicly available epigenetic data from synovial fibroblasts has precluded to assess how genetic variation from the adalimumab-associated GCM impact on this relevant cell type and, consequently, on the adalimumab efficacy in RA. Moreover, recent studies using the single-cell RNA-seq technology have identified new cell types in the RA synovium (112). Future studies incorporating this technology are therefore warranted not only to corroborate our findings, but also to identify new and cell type specific GCMs influencing anti-TNF response in RA.

In conclusion, integrating transcriptomic and genetic data, we have identified a genetic module that is associated with the clinical response to adalimumab. These results provide new insights into the biological basis underlying the differential response to anti-TNF agents and suggest that drug diversity should be considered in the search for treatment response biomarkers.

## Ethics Statement

This study was carried out in accordance with the recommendations of the guidelines and regulations of the Hospital Universitari Vall d'Hebron Clinical Research Ethics Committee and local institutional review boards with written informed consent from all subjects. All subjects gave written informed consent in accordance with the Declaration of Helsinki. The protocol was approved by the Hospital Universitari Vall d'Hebron Clinical Research Ethics Committee.

## Author Contributions

All the authors were involved in the design, analysis, and interpretation of data. All authors revised the manuscript and gave final approval for its submission.

### Conflict of Interest Statement

The authors declare that the research was conducted in the absence of any commercial or financial relationships that could be construed as a potential conflict of interest.
