# Supplementary material for: A Combined Transcriptomic and Genomic Analysis Identifies a Gene Signature Associated With the Response to Anti-TNF Therapy in Rheumatoid Arthritis
Source: Front Immunol. 2019 Jul 2;10:1459. doi: 10.3389/fimmu.2019.01459 (PMC6614444; doi:10.3389/fimmu.2019.01459)
Supplement: Supplementary file 1 [file Data_Sheet_1.pdf]

A combined transcriptomic and genomic analysis identifies a gene signature associated with the response to anti-TNF therapy in rheumatoid arthritis.

Adrià Aterido[1,2], Juan D Cañete[3], Jesús Tornero[4], Francisco Blanco[5], Benjamín Fernández-Gutierrez[6], Antonio González[7], Joan Maymó[8], Mercedes Alperi-López[9], Alex Olivè[10], Héctor Corominas[11], Víctor Martínez-Taboada[12], Isidoro González[13], Antonio Fernandez-Nebro[14], Alba Erra[15], Simón Sánchez-Fernández[16], María López-Lasanta[1], Mireia López Corbeto[1], Raül Tortosa[1], Sara Marsal[1], Antonio Julià[1,*]

[1] Rheumatology Research Group, Vall d'Hebron Research Institute, Barcelona, Spain

[2] Department of Experimental and Health Sciences, Universitat Pompeu Fabra, Barcelona, Spain

[3] Rheumatology Department, Hospital Clínic de Barcelona, Barcelona, Spain

[4] Rheumatology Department, Hospital Universitario De Guadalajara, Guadalajara, Spain

[5] Rheumatology Department, INIBIC-Hospital Universitario A Coruña, A Coruña, Spain

[6] Rheumatology Department, Hospital Clínico San Carlos, Madrid, Madrid, Spain

[7] Instituto de Investigación Sanitaria Hospital Clínico Universitario de Santiago, Santiago de Compostela, Spain

[8] Rheumatology Department, Hospital del Mar, Barcelona, Barcelona, Spain

[9] Rheumatology Department, Hospital Universitario Central de Asturias, Oviedo, Spain

[10] Rheumatology Department, Hospital Universitari Germans Trias i Pujol, Barcelona, Spain

[11] Rheumatology Department, Hospital Moisès Broggi, Barcelona, Spain

[12] Rheumatology Department, Hospital Universitario Marqués de Valdecilla, Santander, Spain

[13] Rheumatology Department, Hospital Universitario La Princesa. IIS Princesa, IIS La Princesa, Madrid, Spain

[14] UGC Reumatología, Instituto Investigación Biomédica Málaga, Hospital Regional Universitario, Universidad de Málaga, Málaga, Spain

[15] Rheumatology Department, Hospital Sant Rafael, Barcelona, Spain

[16] Rheumatology Department, Hospital General La Mancha Centro, Ciudad Real, Spain

* Correspondence: Antonio Julià, Rheumatology Research Group, Vall d'Hebron Research Institute, Barcelona, 08035, Spain. Tel: +34934029082, Fax:+34934034510, e-mail:toni.julia@vhir.org.

SUPPLEMENTARY TABLES AND FIGURES

INDEX

1. Supplementary Tables **3**

1.1 Table S1. Clinical and epidemiological characteristics of the two GWAS cohorts of RA patients included in the study 3

1.2 Table S2. Gene coexpression module associations with the clinical response to anti-TNF treatment at the transcriptomic level 4

1.3 Table S3. Gene coexpression module associations with the clinical response to anti-TNF treatment at the genetic level 13

1.4 Table S4. Gene overlap between the 149 GCMs and genetic findings from previous studies 17

1.5 Table S5. Genes mapping to the biological processes that are nominally enriched in genes from the adalimumab-associated module 18

2. Supplementary Figures **22**

2.1 Figure S1. Principal components of the RA cohort used in the discovery stage of the genetic association analysis 22

2.2 Figure S2. Nucleotide metabolism pathway integrating GCM and SNP information 23

2.3 Figure S3. Epigenetic fine-mapping of genetic variation from the GCM-7 and the cell-type specific H3K27ac and K3K4me1 marks 24

1. Supplementary Tables

1.1 Table S1. Clinical and epidemiological characteristics of the two GWAS cohorts of RA patients included in the study

CHARACTERISTIC	GWAS COHORT RA-1	GWAS COHORT RA-2
**Population**	Spain	Europe and US<br>*(Cui et al. PLoS Genet, 2013)*
**Disease diagnosis**	1987 ACR criteria for RA	1987 ACR criteria for RA
**Molecular data**	DNA	DNA
**Sample size *(N)***	348	2706
**Gender (female, %)**	83,84	73,58
**Age at diagnosis (years, m ± sd)**	42.98 ± 11.46	54.83 ± 12.26
**Adalimumab**		
**Baseline DAS28 (m ± sd)**	5.30 ± 1.04	5.64 ± 1.15
**Responders *(N)***	41	438
**Moderate *(N)***	33	462
**Non-responders *(N)***	20	171
**Infliximab**		
**Baseline DAS28 (m ± sd)**	5.65 ± 1.02	5.94 ± 1.22
**Responders *(N)***	30	289
**Moderate *(N)***	57	361
**Non-responders *(N)***	41	244
**Etanercept**		
**Baseline DAS28 (m ± sd)**	5.71 ± 1.17	5.85 ± 1.28
**Responders *(N)***	46	253
**Moderate *(N)***	52	319
**Non-responders *(N)***	28	161
**Anti-TNF**		
**Baseline DAS28 (m ± sd)**	5.58 ± 1.22	5.79 ± 1.23
**Responders *(N)***	117	980
**Moderate *(N)***	142	1,142
**Non-responders *(N)***	89	576

Abbreviations: ACR, American College of Rheumatology; DAS28, disease activity score before treatment initiation; M, mean; N, number of patients; NA, not analyzed; RA, rheumatoid arthritis; SD, standard deviation; TNF, tumor necrosis factor; USA, United States of America.

1.2 Table-S2. Gene coexpression module associations with the clinical response to anti-TNF treatment at the transcriptomic level

GCM	*P*	GENE#	GENES
**GCM-1**	2,10E-04	21	*SLC25A11,MRE11A,PSMD3,RUVBL2,CLPP,C6orf125,NUDC,DULLARD,C19orf24,DDX1,ZDHHC16,TFPT,MRPS5,IGF1R,PLCB2,RHBDD3,HORMAD1,HPS4,C1orf71,SNX4,STIP1*
**GCM-2**	4,76E-03	61	*UBXD8,CUTL1,C22orf5,TOR1A,C21orf66,DFNA5,EDF1,SOCS4,BCCIP,RAB25,KIAA0406,MLL4,CSTF3,C6orf62,C12orf52,ABCF3,MRC1,ZCCHC3,NUDT16L1,HIST1H4E,ANXA4,OSBPL7,PEO1,GHRL,ACSS1,PPIL2,YTHDF1,DIDO1,MTMR4,ANAPC1,C1orf142,GAGE1,HDGFRP3,GBA2,SFXN2,TNRC15,PPP1R3G,CABYR,RECQL5,API5,TRRAP,CCNB1IP1,ORF1-FL49,HDLBP,ATP2B2,ZNF202,PPP1R10,HS3ST3B1,CDC2L5,MASK-BP3,SLC16A5,KCNMB3,TSTA3,CHMP4B,ME1,DKK3,NINJ1,SMG7,YTHDF2,TNFSF12,RAD9A*
**GCM-3**	7,57E-03	52	*PLS3,ITGA11,CDC14A,ARID1A,LRRC14,GALIG,ZGPAT,DNAJB4,CABP5,SMARCB1,KREMEN1,TTF1,GBE1,NR3C1,CCDC8,MOG,UBE2B,TMEM67,RGS11,PPP1R3C,OSBPL9,RIPK5,ARHGAP27,BNIP3L,MCM3AP,BNIP3,RPUSD1,PCSK7,RESP18,PSMA8,HABP4,AMMECR1,SAV1,TXLNA,GOLGA7,AQP3,C17orf62,CRIM1,AGTPBP1,ATP9A,RAPGEF1,FLAD1,DAOA,CCDC50,TMEM28,RCSD1,RYK,AK3L1,NAP1L1,C1orf59,RGS18,AVPI1*
**GCM-4**	7,65E-03	22	*KIAA1468,ID3,LRMP,SP2,PIAS4,TNFRSF11B,UGT2B7,TARBP2,KLHDC4,ID1,PIGL,TNK2,VEPH1,DUS2L,MBD3L1,ANKRD42,HSPA2,SRM,POU2F2,SLC25A37,FMOD,OR6K6*
**GCM-5**	1,35E-02	143	*C19orf25,APOA4,ZNF264,LCMT2,TGFBRAP1,OAS1,LY6H,RG9MTD3,ACTR3B,E2F3,EIF1AX,RHOQ,EHD3,ADRA2C,DBT,ATP5S,PHF21A,CTAGE6,VPS26B,FAM108A1,NR1D2,RNU33,DMRT2,CCT6B,PCYOX1,BRD3,C7orf36,DNM1L,ARHGEF11,TMEM113,FBXO7,TAIP-,ZFYVE1,EFNA4,SPIN,CAPZA3,KIAA1166,RRAGC,ABI2,RNF170,CYP7B1,BBX,NR1D1,JMJD2D,PCSK9,DNAH10,ITM2A,SYNJ2BP,HNRPA1,C1orf84,C1orf93,ZNHIT4,GATAD2B,PCDH1,ZNF136,STK32B,TCF4,CDC73,PDCD4,OR8K1,TRIM6,C6orf69,LOC153684,CDKN1A,FOXC1,MRPS18B,SIAE,TPH2,TNRC6A,JUN,OR8K3,OR7E24,FGF18,CACNA1C,DIP2A,IRGM,PIP5K2B,TSEN2,PCOLCE2,SH3KBP1,CCDC55,UPF3A,ZNF689,C2orf15,WDR23,TERF1,PCDHB7,TMEM63C,SHC1,ARMCX5,HYDIN,APIP,ZNF641,RPL10,TUSC3,REXO4,CD164,SAMD4B,ITPKA,FDXR,ACOX1,CHES1,UNC5A,MAP4,C9orf85,UPP2,DPF2,ZNF148,ANXA11,RAB3GAP1,RPS20,KCTD12,UBL4A,TAF1,SERPINH1,ZNF197,C10orf67,USP14,SPANX-N3,TAF7L,BANK1,PPP1R2,CCDC25,FOXO1A,CAMP,RAB7L1,ALDH3B2,GCC2,RPPH1,RNF43,SYCE1,VPS54,LIF,GLT8D1,VPS13A,HIF1AN,ISG20L2,KIAA0649,CYP3A5,ACVRL1,MEF2C,LGR6,SMARCC2*
**GCM-6**	1,90E-02	27	*CCDC6,SLC28A2,ZDHHC6,SPATA19,DAZAP1,MRPL22,C20orf85,INCA,CHMP4A,C6orf142,TBL1X,NME2,RNMTL1,TTC13,IFT57,CSTL1,GJB1,SCAMP1,C22orf23,ASCC2,TMEM97,PDGFC,STAMBP,CLSPN,EGFR,KCNK10,MRPL9*
**GCM-7**	2,47E-02	18	*BIN3,OATL1,GALNTL2,SAFB,AGPAT7,DPYD,KIAA1456,RNU26,AK5,PIK3R1,TRIM36,KRT2A,LARP1,ZNF562,SPESP1,MTX2,HIPK1,FAM53C*
**GCM-8**	3,26E-02	119	*USP4,IL23A,HTR4,RSC1A1,UCK2,CD163,ARSB,SDSL,BCL7A,RFWD3,FAM49B,ZNF706,C6orf75,JRK,IMP5,LSM14B,ATF7IP,TREML2,NBPF11,ADRA1A,C12orf46,RPA3,FGF7,CHD9,UBE2Q1,RNU28,CEP290,FAM8A1,ATP1B3,MYH7B,CCDC77,FEM1A,C1orf41,MAD2L2,MRPS35,VPS33A,CARD14,PDCD5,NDUFS2,SLC6A6,SYAP1,CCDC44,F2RL1,NUDT18,C17orf60,BAG5,STX6,ZNF577,LILRB1,MAGEA8,ACOT11,TLR1,ATP6V1H,CHUK,FAM82B,NAP5,THPO,EP400,ARPC4,GTF2I,HDHD1A,GATA5,RSBN1,MKLN1,MRPS12,CYB561D2,SP8,ZNF135,C1orf38,TTLL13,ZNF606,C3orf18,BAT5,SLIC1,CPNE3,PPP2R5C,COMMD2,GTPBP8,CDC42SE1,NXPH3,B4GALNT4,GOLGA8A,MS4A5,GAS6,LSM4,BCORL2,PIAS2,CUGBP2,FHL2,KIAA0753,B3GALT5,APRIN,PHF20L1,CD46,AP1G2,TMTC2,ZSCAN4,KRTAP4-,GPR61,ADCY2,SLC6A4,PFDN4,RDH8,DMRTA1,TBC1D22A,C20orf118,PRR11,PGD,PRICKLE1,MYBPC3,HNRPU,C8orf42,DNASE2,RNF126,NALP13,IMP4,KIDINS220,CDC7,PDCL3*
**GCM-9**	3,33E-02	18	*HUWE1,ASZ1,DDX24,BPGM,SERPINA5,RPL3L,NUPL1,PDSS2,PHF11,ZNF710,CLTCL1,APC,KLHL21,HIP1,MDN1,ALS2CR8,LENG8,C21orf91*
**GCM-10**	3,58E-02	87	*IFNA10,C17orf71,CDC14B,TSPAN17,RTTN,MBIP,ANKRD16,IKBKE,MDS025,SEMA6B,ATP5J2,ANKRD27,TMSL8,EDA,TPD52L3,DVL1,WDR48,VHL,WNT7A,OR52D1,TRHR,KIAA0774,UBN1,CTAGE1,HEYL,HELZ,ARAF,OR10H1,TMED10,KCNQ2,OLFML2B,U2AF1L4,BXDC2,GULP1,GAK,EFTUD2,RBMY1A3P,CACYBP,KLHL8,TBX10,ARHGEF1,SNRPG,ABCB4,SPDY1,PTBP1,APOA1,OR13G1,SNRPN,RBM10,C9orf42,TSNAX,FAM73B,STAC,C9orf86,ZNF289,SH2D3C,TXNDC8,CAPN7,RLN1,CENTB2,ALDOB,ZFAND1,HAL,HBLD2,STK10,KIAA0350,GMIP,WDR18,TMEM106B,CETN1,ADAMTS10,ACIN1,GAB3,PCNP,FAM78A,C1orf171,KBTBD7,PRR7,LGR7,UBAP2,C2orf27,WIBG,MPP5,TNFAIP8,CLDN10,ZNF425,C9orf142*
**GCM-11**	3,70E-02	32	*NIPSNAP3A,ZNF322A,P518,COQ6,SCYL1,SCOC,ADAMTS16,RANBP17,ACTBL1,MOAP1,B3GALT1,DOPEY2,GABRQ,GSTT1,ARMCX6,CPSF2,OR4C6,RNF151,REGL,MORC1,EPS15,MDGA1,RP1,TATDN1,ANKRD6,OR10A6,M160,TIMM50,CAPN10,PIGB,CYTL1,FER1L4*
**GCM-12**	3,78E-02	70	*ATP10D,SRF,SMYD5,ZNF410,CEBPG,GPHA2,GFER,ARRDC1,POP2,CLDN9,NYD-SP14,C21orf100,KLK2,GAD2,OR11A1,FKRP,LCE3A,KLHL7,LOC554223,PPARG,MDK,RAB27B,DBH,GFRA2,LRBA,GPR133,TRAPPC2,PDAP1,WHSC1,EXTL1,TGM5,ZNF516,RCBTB1,LOC284023,SFRS3,OPN1LW,BRD1,RPL39,SHH,H*

			RH3,PCDHA2,OR6C3,UBL5,FLT1,PRLR,LRP5L,TOPORS,KRTAP26-1,CCDC35,ZNF230,OR5F1,EIF4ENIF1,OR10K1,TUBGCP2,LNPEP,IMPACT,CPLX2,TMEM16G,FGF4,NRK,NT5C3,POLRMT,SPAM1,MAPT,CRTC1,OR1J1,EPC1,WDR66,KIAA0232,ANKRD15
**GCM-13**	4,56E-02	24	*AYTL1,PCDHA6,COMP,CNOT4,MAST1,HS3ST3A1,SLC27A4,XRCC3,CAPN2,CHRNA10,UBE2E3,BDKRB1,QP-C,DSTN,AKR1C1,HIST1H2BB,STEAP2,NDUFB11,CRMP1,LSM2,PRUNE,DSCR1,COL8A2,RPL35*
**GCM-14**	5,39E-02	17	*MATN3,THRAP5,NEK8,WDR68,KIAA0103,MTMR7,ADCK5,NACAP1,PTPN23,NR0B1,SLIT1,C12orf40,CNOT10,FKBP9L,MAEA,IRS4,PRKCBP1*
**GCM-15**	5,87E-02	128	*LDLR,LRRC4C,ATP8A2,MFAP5,WDR63,CCRN4L,WDR21A,SART3,STXBP6,CHX10,COL24A1,OR5M8,SLC4A1AP,LEPROT,FAM24A,GPR112,C10orf12,GGA2,ATF3,CALM2,HSPH1,UGCGL2,IFRD1,KIAA0241,LOXL4,OGFRL1,SPATA2,TPRXL,AP2A1,RPL34,ZNF45,RANBP9,HDDC2,EGFL6,WDR73,SYT1,UBR2,ZNF217,SSX1,PSCA,BCAM,CRIPT,NFYA,ARID5A,ARL4C,HYAL2,TWIST1,ALG12,ZNF688,ADCY1,FAM13C1,CTSO,DNAJC5B,ARL6IP5,C20orf123,ADORA3,SPATA5L1,LARS,C8orf40,GOLGA1,TADA2L,MAGEE2,TIFAB,BCL2,PLEKHA2,IFT74,CH25H,GRIN3B,COPS2,IFNB1,ABCA7,CCT4,INSIG1,UBPH,SELP,PDZK1,IL1B,IGSF11,DUSP16,CAD,DARC,RSHL3,PLK2,HIC2,XDH,NFAM1,ZBTB26,CCL26,SLC9A8,C18orf54,B3GAT1,FBXL20,SNN,RBKS,OR9G4,PIP5K3,CCDC16,DUSP2,SLK,CHORDC1,C21orf81,TRIM28,USP11,RNF111,CDYL2,C1orf149,IVNS1ABP,CFTR,GUCY1A2,DHRS4L2,S100A7L1,NXPH4,MYOHD1,TTLL4,CUEDC1,KAL1,USP37,APLP1,ZA20D2,THBS1,NME5,DPYSL4,PARS2,SLC7A2,RAPGEF4,LAYN,LANCL2,OR6C68*
**GCM-16**	6,32E-02	31	*ZNF10,URP2,SMAD5,CHCHD1,C2orf4,ATG7,ABCA5,SERPINB8,TRPV2,ZNF510,PUM1,RPS6KB1,FN5,UBE2S,FBXO21,CARD9,PCBP2,GNG5,C6orf55,MFSD4,GLTSCR2,EIF4E2,PSIP1,HPRT1,SDC3,EIF4B,RCC1,SLTM,FNBP4,SNX8,RBJ*
**GCM-17**	6,64E-02	244	*WDR59,CCDC7,CNDP2,G3BP,SVIL,TNPO2,SGPL1,ACTR2,DHX34,MAB21L2,TSEN34,ACTL6B,SOX15,ST3GAL2,RNASE12,SMARCA1,DEPDC6,UNQ2541,GPX3,ZFYVE26,ATP6V1D,MFAP3,ZXDC,ZNF563,TCEA3,AIM2,AVEN,CAMLG,MLZE,IL11RA,TCP11L1,PACS2,TTC9C,PAPSS1,SEMA4A,GSTA4,PSME2,POU6F1,TLR8,DCAKD,RBL1,YAF2,HNRPH3,PRIM2A,C14orf151,CDK5,ERAL1,YPEL3,RABGGTA,LCP2,C6orf146,TOR1AIP1,FYN,EVI5L,DTX3L,LSM12,NRAS,YARS2,MAPK3,TRHDE,BAK1,VAPA,SMYD2,EPN2,IL6,GSTM5,CA12,TSSC1,TAOK2,GNGT2,AP2B1,HLA-DPA1,TMEM80,CEACAM3,FSIP1,PCDHB12,CPO,ZDHHC5,ZNF449,HPS5,SFRS8,CATSPER2,NHLRC2,ATG3,RAB3D,HOXA5,ABCA3,C1orf155,SLC2A6,CITED4,NUP50,KCNJ14,SP100,ZNF200,GRB10,C12orf5,IMPDH1,OVGP1,MYH8,HSD3B1,SLC39A8,HLA-DRA,AGL,NBPF15,ZNF274,LYSMD2,IL15RA,SLC8A3,MLSTD1,PEX5,MYB,IQCG,BST1,GLO1,ARP10,CCBE1,PHF2,PITPNM3,MORF4L1,STX17,SEC22L3,ZNF96,ADAMDEC1,VAX2,CRY2,C5orf16,SPIRE1,ATP6V1A,C10orf4,CHCHD4,MCFP,TNFSF13B,FAM58A,ACOT7,TAGAP,ZNF629,CENTG1,MOXD1,XTP3TPA,SQLE,HMBS,TIMM23,RASSF4,STAT5B,HSF4,TMPRSS3,PRPS2,LTC4S,VEGFB,HSPA8,OR6C74,CNTNAP5,MXD4,TRIM25,MECP2,PCGF2,LRP8,KIAA0831,FAM38A,SLC25A15,RHEBL1,RAB24,PDCD1LG2,ATF6,PHLPP,C19orf33,HTR3D,CYP20A1,PCDHB4,GNRH1,SYT17,ECT2,FAM21C,KIAA0179,RUTBC1,IL12RB1,SYNGR1,RGS19,SLC5A10,SLC27A2,DCUN1D2,ERICH1,C9orf52,SDCCAG10,DDX49,TCL6,TOMM20,IQWD1,PLEK,TSPYL2,CCL18,DNAJA1,CANT1,IPO4,DDR2,YWHAZ,HNMT,SLC39A11,VRK2,SH3GL3,NUDT5,GOLGA2,TMEM38B,SEC63D1,LTBP4,EZH2,ZDHHC18,COPS5,LIX1L,PPP2R3A,RAVER1,HIP2,WHSC1L1,CFD,SGTA,FAM112A,ADAMTS3,TMOD3,RASGEF1A,RPE,GUCY1B3,A4GNT,SF3A3,ZFYVE21,ANKRA2,EIF2C3,HEM1,PLD2,ASGR2,TAGLN3,PANK4,PPM1A,PINK1,PEBP1,HLF,GNA14,UCK1,PITPNM1,DOK6,PRKCG,DIRAS2,ZNF680,ZNF259,CLEC6A*
**GCM-18**	7,32E-02	16	*OR1E1,SETD8,TAS2R40,PPFIBP1,STEAP1,CMYA3,GJA1,CPT1A,SYNJ2,DEFB32,ABCD3,SOX11,CDK5RAP1,PER2,ZNF41,AADAC*
**GCM-19**	9,39E-02	93	*SORD,DNAJC5,SNX12,SLC4A8,CLCN1,MMP14,ADAR,ABHD8,NUDT21,GEMIN7,LMLN,CYP4F11,OR4N5,KRTAP10-10,WBP11,OR9Q1,CASP8AP2,VARSL,MTIF3,TM2D3,SSX2IP,WDSUB1,C1orf58,MARCH8,ABCC2,ZNF291,ZNF664,SCAND2,NKIRAS2,VAMP8,OR5M3,KIAA2026,UNC5D,LAPTM5,MYO5A,C14orf93,C9orf90,MAN2B1,H2AFV,ACRBP,CHMP6,CAPN1,C11orf45,SCGB1D1,SLC13A5,NFKBIB,MED9,TPP1,ARRB1,LRRC33,C9orf97,DNAJC15,KRT17,CD33L3,TESSP2,PIWIL4,TNFAIP8L2,PDPR,SLC39A5,NIPSNAP1,ADCK2,RTEL1,RHCG,PIK3AP1,RAB35,BIRC5,MYST2,GLT25D1,NPC2,NEXN,OR13A1,PAK3,TNP1,RBL2,USP42,WNK1,C20orf55,SF3A2,RPUSD3,TBX19,ZMYND10,C14orf94,DSG2,PQLC1,AYTL2,TMEM127,FBXW11,CD4,MET,TMEM86A,TULP4,PIN1,LUZP4*
**GCM-20**	1,02E-01	30	*RNU42A,NEUROD6,P2RY14,PCDHGA5,C3orf42,ZNF37A,KATNA1,OR8S1,GRIK2,C1orf185,NANOS2,TRIO,DKFZP586H2123,OR3A4,DGKZ,PQLC3,IL13RA2,CEACAM6,SCNN1G,SEPX1,SERPINA7,ZNF530,FAM70A,SMOC1,PRR5,DPP8,HDHD3,ZBTB10,KRTAP5-7,KATNAL2*
**GCM-21**	1,13E-01	19	*INSL5,MARCH3,SESN3,GPR35,MRM1,NHS,INSM2,MC3R,RAD54B,KLHL18,PPP1R7,FOXP3,ASH2L,FOXO3A,RBM28,RRAGB,GLIS1,TAS2R7,ZNF660*
**GCM-22**	1,28E-01	112	*FRAS1,GPR65,MASP2,WHSC2,KRTHB2,CEP1,MST1R,BRS3,TPH1,ENTPD5,ZNF439,PLXDC1,FMO3,LSM5,CYorf16,MULK,PTOV1,HIST1H2BM,THBS2,COL1A2,TSPAN11,IGFL1,HIST1H3I,SERHL,CTSG,SLC5A5,MLH3,CA7,WFDC3,LEPREL2,AIM1,NEDD4,ANTXR1,HMCN1,CXorf38,ARGFXP2,CPA3,GNB4,ROR2,LBX1,KIAA1217,OPCML,KIT,IGSF8,HIST1H2BF,COL21A1,MAP2K1,NLGN2,DLX5,MRPL23,PDGFRL,RTKN,LYG2,COL14A1,VPS29,GPR155,OR6C6,GDF11,MTBP,LSM3,ADCYAP1,KIFAP3,TMEM98,NOX4,ELOVL4,BSG,FILIP1,SIX4,DOT1L,THRA,GPR15,C9orf24,CER1,MS4A2,HRG,RDS,SCG2,SMOC2,CYC1,CRY1,PRSS23,TMEM119,GOLPH2,CARKL,AFAP,GLI3,SPAG17,ZNF77,TM4SF11,ZNF38,HAMP,PRAM1,BNC2,TMEM16E,SCGN,HIST1H2BC,FBXO36,ZNF414,MLL5,NDUFA7,TNFRSF19,MRPS7,SLC35E2,PGCP,PMS2,MASTL,NPTX2,F*

			NDC1,ZNF677,SLC26A3,PKHD1,RAI14
**GCM-23**	1,34E-01	35	*NEUROD1,SPRR2F,FOXN4,TRIM52,PAGE4,XYLT1,MS4A12,ELA2A,SGK2,SERPINA9,DPPA3,COL4A5,VPS53,GALP,DAZ2,TCP11L2,SETX,C12orf30,PML,SEC14L3,GLRA1,RANBP2,DMRTB1,PNPT1,CBX1,RNASEL,SELV,KIAA0261,PKDREJ,NYD-SP21,UCN3,SULT1C3,C4orf15,ELP4,HCG18*
**GCM-24**	1,35E-01	32	*PTTG2,MYO1B,ZNF419,CCR10,C1orf128,ZNF8,ZNF501,ACY1L2,INPP1,SLC26A5,RPS3A,GRIK4,PVRL1,MDFI,SPHK1,TRAIP,CRYBA4,SMOX,ACY3,EEF1D,WNT5B,TRPT1,PPP1R3E,TUBA2,CCDC24,FTSJ3,GBP7,B3GALT2,MLNR,DSP,GAD1,CNTNAP4*
**GCM-25**	1,36E-01	33	*XYLB,AP1S3,EML2,TMEM46,C14orf72,SNX22,LY6G5B,EVX1,KISS1R,MAGEA2B,DPEP1,CXorf33,LRP2,DCTN5,CATSPER4,SETD3,GJB4,NXPH2,GH2,TIMM13,BCL2L11,PDIA3,PCDHGB7,LRRC50,LRRN1,OR10G4,FAM92B,ILDR1,HMGB3,CALML4,DDO,RYBP,BCAS4*
**GCM-26**	1,38E-01	40	*NOS1AP,PRSS27,PDK4,VTN,NR4A1,TEKT2,CACNG8,CLDN6,LRRC21,CYP11B1,CYP2A7,INT1,ZNF554,APOC1,NDUFA3,TMOD2,LDHAL6A,RLN3,NPBWR2,C9orf14,C15orf2,ZNF580,KIR2DS4,PEX6,SAPS2,YWHAQ,ISL1,CCDC72,ROPN1,KIAA1946,COL25A1,LRRC48,KIN,KCNA2,KRTAP1-1,CLDN19,NDUFS6,C17orf56,OR4S1,PSKH2*
**GCM-27**	1,39E-01	37	*SLC26A10,LGALS3BP,MAP3K14,RHOU,RPL10A,TCEB3,KRTAP4-12,DGKA,CES7,BTK,PLCD4,RANBP3,MPG,BST2,CYP4Z1,FBXO33,MAG,LENG9,HBE1,ALPI,PKD2L2,HMX1,CAV1,ITGB6,FERD3L,WIPI2,GTF2H5,KIF17,LIMD1,FXR2,PEX10,GAB2,SELI,DCUN1D3,UXS1,LRRC8B,TK2*
**GCM-28**	1,42E-01	15	*ATG4D,FAM112B,THAP10,NGFR,MRPL55,MAT2B,CXCR4,GATS,TDRD10,H1FX,C17orf73,HSD17B3,PCDH12,UBE2V1,ASMTL*
**GCM-29**	1,47E-01	210	*STARD10,RNU21,IDS,BCL10,FRAP1,ACP1,PREI3,OR8D2,MEF2A,EXOC6,TAS2R39,SLC25A26,PFDN1,ZNF18,LARS2,SPG21,OBFC2A,SPACA3,C9orf72,TREX1,STAC2,C17orf81,ALOX5AP,MAST3,STAT2,GMPPA,TNFRSF8,BCL9,DEFA6,GPR82,RNMT,FARP1,TNFRSF14,RBMS1,GPR12,CPNE1,GIMAP8,PEMT,TTTY4,CLIC3,C7orf26,DHX37,HHIP,TRPC6,USP22,TRG20,TRPV1,SFRS14,PDCD10,C1S,COX4I1,MCM3,CGRRF1,BTF3,SEPT10,TMEM126B,PPP1CB,ST8SIA1,CD58,CAPS,RIC8B,TUBGCP6,ODF2,GPR42,FANCD2,NTN1,KIAA1191,PHF17,AMICA1,WNT16,DDX19B,SEC24B,ABHD11,JMJD4,DDX6,C1orf96,SCP2,YIPF7,BAZ2A,BAX,C20orf70,CEBPE,DGAT2L4,PPP3CA,TAC3,SDFR1,TOR2A,STRN3,DTNBP1,KLF4,LCN9,TMPRSS7,PSD2,C8B,CRISP1,ZNF92,DBR1,SCTR,CCNK,PCDHB8,B3GAT2,ADH5,LBA1,C21orf119,IPO11,GPR20,DCP2,BCAS2,NAGK,OTUD6B,PYGO2,RAB28,DHX16,APAF1,RPTN,IL31RA,SLC11A1,ZSCAN2,GNMT,RIP,DNAHL1,MUS81,HS3ST1,CPN2,CDC27,CDC25B,BAG1,C14orf24,HHLA1,C10orf26,FOS,SOCS7,HIP1R,PANK2,SLC25A16,CAMKK1,ERG,ZC3H3,PCDHA3,TMEM14A,NEK2,SPATA9,PITPNC1,OTOA,MICAL1,KRTHA1,OCIAD1,SEH1L,NSUN5,RAFTLIN,PRPF40B,THYN1,RBP5,CHMP2B,CRYZ,FBXO22,USP53,PTPRZ1,RPL10L,LOC441242,RAB37,GNG12,CALML6,PBX4,METRN,MLR2,POLE,CMAS,TNFSF10,ZNF17,TERF2,SATL1,LRRIQ1,ASAH1,LRRC30,BTF3L4,DHRS7,FAM92A1,RPS10,RASL10B,CAST,CHMP1B,PRKCH,GABRG2,CPT1B,DCTN6,EME2,XRCC2,FBXL10,RFX1,RAD52,SPTLC1,C2orf28,SF4,SKP2,FVT1,C12orf38,LYZL2,NF2,CLEC10A,LYCAT,SLC25A21,C1orf105,AQP1,FUNDC2,FBN2,PTPNS1L3,TATDN2,PTP4A1,TAAR6*
**GCM-30**	1,47E-01	29	*QIL1,CCDC74A,ASB9,STAG1,HIST1H3D,ATP4B,C6orf84,CLEC2L,PAPD4,SMNDC1,PBXIP1,CKLFSF4,ANP32B,TROVE2,TSPAN4,LCE2C,RNF146,MYO3B,ANKFY1,TMEM118,MPZL1,MRPL12,LYSMD3,SLC9A4,CECR2,TIPRL,GAS2,KLK5,TBK1*
**GCM-31**	1,52E-01	40	*CNOT1,SPEN,PECI,TSPYL5,KCNB2,LAMB3,R3HDM1,MSH3,CXorf20,KIF1B,RGC32,GGPS1,DSCR4,OR8D1,PSMD1,MLL,CLDN23,IFI27,LGMN,KRTAP19-2,APOL4,GPR128,MXD3,PPM1G,CDKL3,SENP1,SFRS2,NR2C1,RFK,DAK,BMF,ST6GAL2,ZNF224,IL21R,CDH23,VN1R5,PSME4,C5orf15,FRMD4B,HNRPD*
**GCM-32**	1,75E-01	23	*SYNGR4,DDI2,KIAA1205,DTX4,TSKS,DNAJC12,SLC6A2,VSIG2,KRTHA7,MAX,FAM59B,HTR1D,CSNK2A1,ABCC13,SPSB1,CUEDC2,PARP3,C20orf45,TMIGD,CPSF3L,DDC,C16orf5,DNAJA5*
**GCM-33**	1,79E-01	17	*SMARCC1,TEX13B,HERC1,ZNF692,KIAA1467,ITGB1,C17orf77,CHRAC1,HPSE2,PNMA5,MFAP3L,C10orf13,APOA1BP,TAS2R43,RAX,ATP13A5,HEMK1*
**GCM-34**	1,81E-01	66	*ZSWIM4,ADC,C12orf34,ARRDC2,PHF1,RSPO2,VPS39,C6orf108,HSPC176,ZFP41,OR1J4,SLC44A3,CYLC2,FAM19A5,LMX1B,ZXDA,GLYCTK,KRTAP6-3,MSX2,NALP12,AFM,RAMP1,NDUFB10,TAS2R50,EDA2R,C1orf2,CHERP,DPM3,ZBTB2,RAB17,LYPLA2,OR52K2,PRSS3,HSD3B2,MCTP1,ATP5G2,FSCN3,NGEF,CIB4,RHPN1,PCSK4,ANKRD24,ALS2CR19,TAF6L,HCN1,CASK,SLC9A11,POPDC3,AIM1L,OMD,CBS,HAK,TRIM60,OR2A5,RPS28,C17orf61,ATP5D,KLHL4,GPSM1,ORAOV1,ASB1,SLC30A1,MLLT11,TRIM51,WISP3,OR13C5*
**GCM-35**	1,85E-01	24	*TREML1,IDI2,HLA-C,HSA9761,SLC34A3,RHPN2,C16orf3,TMEM121,TG,ZNF549,KCNF1,MRVI1,C10orf9,CYP19A1,STIM2,GCHFR,IL16,TPD52L1,DJ122O8.2,ABCG8,TAAR8,RNF20,RBM4B,FAM82C*
**GCM-36**	1,89E-01	31	*KA36,PHIP,RNU38A,CYP26B1,LKAP,ARL16,MTUS1,NPLOC4,AFF3,OBFC2B,HMGB1,HIST1H2AD,PLCL2,ECE1,PPGB,DNHD1,TEP1,C16orf28,C1orf33,SUMF2,NPTXR,STAT3,DENND1B,C14orf130,SENP8,ZFYVE28,KCNE2,DKFZp686O24166,EXOSC2,SH2D4B,SLC22A13*
**GCM-37**	2,19E-01	25	*ZCCHC16,FAM102B,NPM2,RPA1,CYorf14,CRHR1,ATP12A,PHF15,GRINL1A,SPANXC,IL13RA1,AGPAT4,SPRR2A,GCAT,CAPZA2,FGG,IFT81,MAGEB4,C7orf13,RHBDL2,MRS2L,FBN1,RFX2,MDH2,RPS2*
**GCM-38**	2,35E-01	18	*C20orf39,RNPC3,MOCS3,NEUROD2,CXCL13,LDHD,SDC2,LRP11,SERBP1,MGAT5B,OGN,C6orf32,RASSF2,SELL,VNN1,ZNF433,RPP40,SLC2A7*
**GCM-39**	2,39E-01	16	*SLC30A9,SF1,UBE2Q2,TMEM77,BAG4,ELOVL7,CD24,VPS36,TP53BP2,CYLD,PHTF2,C14orf92,FOXF2,MKRN1,PKN2,CPLX3*
**GCM-40**	2,43E-01	21	*GDPD1,ARNT2,TSC22D1,SERPINB3,IGSF4C,OR51M1,COL8A1,RHD,ATPBD1C,ARMCX3,FBXO32,SLC25A25,MAK,C9orf11,USP30,SLC39A10,PGBD3,PRAMEF10,VRK3,PLCXD2,ARL10*

**GCM-41**	2,45E-01	45	*LSAMP,ADAMTS17,C11orf38,LMBR1L,FBXW8,CAV3,RBP1,KCNH2,ELAC1,USP20,PAIP1,ABRA,ZNF187,ZFYVE19,ZMYND12,ANXA9,ZNF626,RCN2,KRT23,DBF4B,WNT4,SLC22A14,C11orf37,HMG20B,HDCMA18P,SULT2B1,MGAT5,PXMP3,RPS13,HUS1,TECTA,ZNF703,DLG4,C20orf173,GSTA3,FGL2,ABI1,C6orf106,FURIN,JMJD2B,DICER1,GOLGA2L1,PRPF39,PSCD2,KRTHB3*
**GCM-42**	2,48E-01	18	*CACNG3,RNF14,KIAA0556,TMEM89,FBLN1,LOC339529,ATP1B1,GNRHR,CDSN,TRIM67,IL1RL2,PH-4,PUNC,TTC5,CPNE8,FKBP2,GALR1,BDNF*
**GCM-43**	2,63E-01	27	*TTN,CTTN,TRMT5,KCTD19,DUXA,CRYBA2,LRRC37B,ACVR2A,NFASC,SMTN,HIRA,ZNF84,FAM53B,PAX3,FAM48A,ZMYM1,BTLA,KCNG2,C17orf32,PLXNA2,SLC26A7,NTRK3,PRRX1,AQP2,IGSF1,CEACAM1,DYNC2LI1*
**GCM-44**	2,65E-01	19	*WNT1,IL28B,CREB5,DNAH11,EXOSC8,CDIPT,PTPN3,TNFSF18,TP53AP1,SLC6A3,KLF2,KPNB1,IKBKAP,TGM3,FAM69A,REC8L1,ARL4D,HIST3H2BB,DEFA4*
**GCM-45**	2,66E-01	51	*OR51A7,DNAJC13,GRIN2C,ACP6,BGN,PDZD6,TBL1XR1,RILP,KRT24,ZNF43,ARMCX2,ZNF334,ZCCHC13,ITGA2B,CCDC11,ACSBG2,TMC1,C14orf162,KLHDC6,OSR1,CPS1,KCNS2,MDH1B,C6orf165,EYA4,SLC37A3,ZNF605,TUBB1,C10orf79,LMX1A,SNAP29,GGTL3,C14orf44,GABRB3,PITX1,RSPO3,SART2,GFI1B,SLC7A8,KLF8,DNAH9,CASC1,ERBB3,KCNAB1,KRTAP5-11,COX11,MAPK7,CCDC74B,NAP1L5,DEADC1,PF4V1*
**GCM-46**	2,69E-01	40	*TMEM9B,KRTAP10-4,SLA/LP,MT1G,GYPE,CRLF1,SLC41A3,DISC1,DNAH8,DLC1,ISL2,UBL4B,TUBA4,KDR,GRIK1,SNX2,SH2D1B,NCR1,PNMA2,SH2D4A,MICALCL,PDE4C,REXO2,IL1F7,OR10Q1,AQR,RASGRP4,MSTO1,DLGAP4,DOCK4,GP9,MEGF10,PLEKHK1,FGF8,USP18,TYSND1,IGSF4B,DUSP7,ADSSL1,HOP*
**GCM-47**	2,72E-01	16	*ST3GAL5,FAM111B,ABCD4,ITLN2,MOBP,C10orf99,PPP2R2D,CDC5L,SUPT3H,TDP1,OR11H6,CUL2,STK11,C3orf52,RAB41,ASTN*
**GCM-48**	2,92E-01	22	*DKFZP434P211,RPL35A,ZNF592,HKR3,RGS9,ITGA9,CD3EAP,WNT2B,CYP2C18,C20orf135,B3GNT3,KCTD18,METTL5,GPR154,AAA1,SMG5,AMPD3,CC2D1B,FES,CCHCR1,TXNDC4,CTDSP1*
**GCM-49**	3,07E-01	16	*CAMKV,GAL3ST2,RTP3,NONO,SLC6A20,MAP3K8,ACE2,GJA5,FAM5C,PART1,SOSTDC1,CLEC2B,RAE1,PLSCR2,MESP2,NPPC*
**GCM-50**	3,13E-01	16	*SGNE1,REST,WFDC13,C9orf78,AMY1A,CALCA,C6orf114,CG018,GTF3C2,C8orf35,KIAA0892,HMG20A,DNAH1,VPS35,FLRT1,PGBD5*
**GCM-51**	3,18E-01	46	*TRIAP1,PGLYRP3,C10orf130,ANXA6,RAB22A,SEPP1,WWC1,TKT,C14orf101,COX7B,CSN3,PRR6,ITGAX,MMP9,DDX4,PSMF1,BTNL8,DKFZP434L187,PCDHB9,ICT1,RASSF8,MAN2B2,RRH,PSMB6,C21orf2,EEFSEC,SLC24A4,ADFP,SLITRK1,TNFSF14,PIGA,IL1RN,OR10X1,LOXL2,CAPN14,ASCL4,MTMR3,HSF1,ASPH,XKR5,TBC1D21,FSTL1,SUOX,CST6,BRMS1L,TM7SF4*
**GCM-52**	3,32E-01	45	*CD200R1,AQP9,TGIF,TAPBP,DCDC2,AANAT,S100A12,FOXH1,ARHGAP12,ABTB1,COL6A2,SOD2,OR2T3,CALR3,GEMIN6,MAGEC3,FXYD2,CXCL6,PDCD6,AGXT2L1,OR8A1,GPR83,RFX4,MCEMP1,LAD1,EDD1,LETMD1,ZNF114,COQ7,LITAF,CCND3,C1QL1,OR2M7,LILRA5,ACSL6,KIR3DL2,ASNA1,CLEC4E,GHRHR,ECGF1,RPL32,WDR77,DEPDC2,FRAT1,NALP2*
**GCM-53**	3,36E-01	35	*RRS1,SIRT5,BZW2,AKR1C4,DPRXP4,PRR3,ADAM11,TRIM54,GSTA1,MAPKBP1,C12orf61,CLEC1B,SETD1A,IHPK3,ZNF548,A2ML1,CBX6,EXOSC6,CLEC12A,MRPL11,CITED2,TLR5,RABL5,WDR5,PTK6,TMEM41A,SLC17A3,UHRF2,EIF2S3,KCNRG,CTRC,UTRN,RDH13,ALDH1A3,SCARA3*
**GCM-54**	3,40E-01	18	*GNB1,COMMD7,OR5H1,ATP6V1G2,UMPS,TAF7,SUHW3,TRIAD3,AP4M1,C6orf64,KLC4,AOC2,ABCB9,CDCP2,PRKG1,ZNF556,OR6A2,IL1RAPL2*
**GCM-55**	3,40E-01	23	*GTDC1,SLC9A9,DPYS,AMBN,FRMD5,SPATA6,ALDH3A1,CLEC12B,RASD2,HSP90AA1,AK2,DCHS2,ABAT,ANGPTL6,NPC1L1,C14orf45,CGREF1,THEDC1,C1orf176,RHBDL3,CBLB,RPS23,RNASE13*
**GCM-56**	3,41E-01	53	*KCNQ1,OPN4,ALOX12,LIN41,C12orf58,UMOD,TRAF5,ARL13A,ITIH2,ICEBERG,SET7,ANKRD1,KRT14,MRPS21,TMED8,PDCL2,APPBP1,NKX6-1,TNFSF7,CCL8,COL13A1,ITPA,GAL3ST1,SNAP23,LOC642929,NCDN,SPATA16,CTNND2,GUCY2F,PCDH15,TEX12,HIST1H4G,OR2L1P,GPC5,PPP1R8,ITSN1,MUC4,TNIP3,FAM14B,CLUAP1,FOLR1,TMEM34,OR13C3,CXorf48,ODF4,ST7OT2,PCDH11X,EIF2S2,SEPHS2,GBX2,SHD,RNF11,CASZ1*
**GCM-57**	3,44E-01	23	*SGCB,ADAMTS5,PDCD8,MGST1,HIVEP3,CTNNA2,RUNX1,OR4K13,TRIM35,FOXC2,PRKCABP,ZSWIM3,SPINK7,C1orf188,FKBP8,CA9,WBP2,PARP10,ZNF408,ZNF124,C9orf89,B4GALT2,SLC7A3*
**GCM-58**	3,53E-01	49	*AKAP4,VAMP2,DMD,C16orf56,T2R55,SFRP4,NOS1,MBP,BRD9,NRG1,C10orf125,ACPP,TTTY10,C3orf19,BMP8B,MARCH4,DEFB129,PHACTR1,EIF2C4,NUTF2,TCHP,PLTP,PYGO1,ZNF214,S100A2,MASS1,ADAM2,LHPP,C9orf29,EIF4E,PTPRG,FOXJ3,AKAP12,MESDC1,PIPOX,HDAC3,NR2E1,MSLN,CDH16,CARM1,STAM,IFIT1,C6orf103,ADD2,DSC2,WNT2,PTPRO,LIPT1,RAB11FIP4*
**GCM-59**	3,54E-01	218	*POLD4,TRPM1,HS1BP3,C1orf27,C14orf156,IL8RBP,TTC7A,KCNMB2,ELF5,SCUBE3,LOC441155,OR10H4,IDH3G,MIDN,PYY,GNPNAT1,SIRT2,SLC10A3,FAM32A,NCOR2,DOK2,THAP9,ANKRD13B,OACT1,PHF13,CGI-38,MRPL33,ARHGDIA,ZNF313,LRRC25,LSM7,TYROBP,AP4E1,PRIC285,GHITM,TAF5L,COX6B1,TRIM41,VEZT,P15RS,RAB39B,IRXL1,ATP5H,CECR5,MFSD5,FCAR,FHOD1,KCTD13,DEFB106A,GNB2,NTE,UBE2A,RPAP1,PSMD8,BRWD1,FGF3,C1orf76,BAZ1B,HK3,COL4A4,USP5,ATP6V0D1,C22orf25,HGS,CASP1,MMP17,MGRN1,POLR2G,LY86,APH1A,MRPS18C,CGB1,DNAJC14,NRGN,KRTAP9-4,ASPSCR1,SF3B4,ANK1,S100A11,SLC6A5,OGFR,MBNL2,TMEM116,IFNW1,C1orf103,IDH3A,ITGB4BP,UPB1,KRTAP19-7,CD33,KIFC3,SAC3D1,ARFRP1,BCL3,ATXN10,TIMM8B,PLEKHQ1,ASNSD1,ARHGEF10L,LFNG,CCDC22,TTYH3,DRAP1,ZXDB,N4BP2,PHF7,RAB5C,SH3BGRL3,SLC39A1,ACSM2,C6orf89,ADAM12,WDR58,DEFB108B,PRMT8,HDGF,ZNF181,NUP188,RNF2,SLC6A11,SEMA4C,PCDHB14,LACRT,ATP6V1F,FZD2,TSPAN3,DMN,MPV17,PCAF,RPL31,HSPC049,NUDT1,SELO,NUP43,PTRH1,RPS21,NDST2,SIAHBP1,COPS6,IER5L,FFAR2,NOX5,ZNHIT2,CCDC41,AOF2,ANAPC2,EXOC2,PRSS8,STK11IP,GPR146,TRIM22,GNPDA1,NOC4L,CCDC38,HNRPAB,LETM2,CLK1,ARS2,DRG2,RPL27A,C6orf205,MYO1F,EXDL2,LTBR,*

			KIAA1409,C2orf24,DPEP2,PECAM1,MSN,NEFH,REPS2,RENBP,C1orf85,MVP,C2orf17,UBE2D1,RASSF5,C16orf7,RPS6KC1,DNTTIP1,CCNG1,TBC1D10B,BAIAP2,NDUFB4,CSNK1A1,ZBTB11,FBXW9,RSAD2,SEC6L1,PDCD7,PCGF6,PPA1,SPANX-N5,PNPLA1,GNAI2,LYPLA3,RNF130,RBMS2,KLHDC8A,CD99,WDR81,CORO1B,AP2S1,TOLLIP,PSTPIP1,SSX8,SULT1A1,UNC5B,C18orf22,LYL1,SPIN1,CSTA,KIAA1602,IMPAD1,POLDIP3,STARD3,C1orf164,IL4R
**GCM-60**	3,69E-01	14	*TPP2,MPP7,THAP5,ORC5L,SOCS5,SPRY1,HERC2,CLEC1A,ACRV1,RB1,MGST2,C20orf74,PRPF4,GFOD1*
**GCM-61**	3,77E-01	24	*HMX3,OR5L2,SERPINA6,KCNA6,FOSB,OR51B2,MPP2,SORCS1,DUS1L,SDS,TEPP,WDR76,OR8H2,NME7,ALS2CR4,DYNC1I2,SLFN5,TBC1D19,EML5,NA,KIAA0804,RUNX1T1,PIK3R3,NHLRC1*
**GCM-62**	3,78E-01	25	*COL5A3,ZNF347,KCNJ8,NOTCH1,TFAP2E,LMBRD1,IMP3,ZNF9,SIPA1L1,OPRM1,C7orf34,LECT2,RNF17,SEMA3F,MOV10,COL4A2,COL4A1,RCN1,FMO1,DHRS1,TM2D1,RPRC1,GH1,HIST1H2BL,NOS3*
**GCM-63**	3,89E-01	43	*RNF34,OR7A10,ARMC2,DNPEP,POLR2C,SMARCD3,WNT9B,TMC4,OR2AK2,FAIM2,KCTD11,OR2D2,GPSN2,NFATC1,FAM73A,ALPK3,DNAH5,RAB36,PTGIR,CELSR2,MLC1,AGXT2,TMEM125,MED12L,PLA2G2F,KIAA1641,SMAD7,CAMK2A,KCNJ1,RCBTB2,OR5D18,FAM26A,C20orf112,F9,PFKFB3,DHCR24,GCNT2,IGJ,TRIM44,AMPD1,RNF113B,OR52E2,FHL3*
**GCM-64**	3,90E-01	40	*KLC3,ESSPL,CCDC47,KCNJ15,MAGEL2,EID3,C12orf12,LGICZ1,BPNT1,BFAR,HCTSL-s,OR2AE1,RCHY1,TLK2,C8orf71,SDCCAG8,AKR7A3,LGR5,ZNF435,MANEAL,UNC119,OR6N1,ARNTL2,DIO1,COCH,HTR2B,C10orf72,FAHD1,GRK7,GPS1,C14orf54,SELK,MRCL3,ABCG1,CALML5,C3orf60,OR4L1,OR2T12,ARTS-1,HNRPF*
**GCM-65**	4,00E-01	24	*OVCA2,DLNB14,GYLTL1B,MAMDC4,DALRD3,SDC1,ERMAP,KIAA1958,ZNF507,C21orf90,OTP,CCL13,EDARADD,ERV3,LRRC39,TRAPPC6A,FKBP4,C3orf35,LARP5,CACNA1G,ZFX,KCNE3,LZIC,AHSA1*
**GCM-66**	4,03E-01	18	*PDZRN3,SLC30A3,C10orf111,DPPA5,RNU83A,LYNX1,AXIN2,MID2,ZNF434,PROC,SUNC1,UNQ467,STARD6,DYRK3,KRT18,CDH15,NCOA6IP,MAP4K4*
**GCM-67**	4,03E-01	24	*ORM1,ORM2,UTS2,YWHAE,NTSR1,RETN,CXorf26,C9orf74,PTPN12,HLA-DRB5,F2R,ELF3,LGR8,JAK2,SPINK5L2,SULT1C1,HLA-DPB2,ARSF,CNNM3,NFATC2,ALDH1B1,PPP2R3B,NOS2A,TSPAN10*
**GCM-68**	4,09E-01	30	*HMGCLL1,PGRMC2,DMBX1,SOCS2,ADRM1,STEAP3,ZNF519,PCCA,ZIK1,KIAA1199,SERPINE2,HPD,RAB11FIP2,TIMP1,OTC,LAT2,CSNK1D,LANCL3,GSK3B,ACTL7A,TUBA6,ARHGAP11A,BCKDHB,PFN4,SPINT2,ZFP106,PILRB,OXCT1,TMEM35,CDH19*
**GCM-69**	4,12E-01	32	*HLA-DRB1,FGF2,UXT,HIST1H2BO,TRY1,OMG,DKK2,C8orf34,UGT1A6,LOC643201,SLC5A7,OR8G2,RTN4,FBL,PHOX2A,OR7D2,KIR2DL1,TRAF6,ZNF132,KRT13,PLA2G1B,SNTG1,SERAC1,CPM,MAGEA4,KPNA3,FRMPD2,KIAA1279,C3orf1,NUDCD1,FAM40B,MUSK*
**GCM-70**	4,25E-01	15	*CART,NALP9,ANUBL1,ZNF560,POU4F2,TGIF2LX,FBXO9,ZCCHC10,LOC643406,NUP155,ANKS4B,JMJD1B,NYD-SP18,FSD1NL,CCDC52*
**GCM-71**	4,29E-01	45	*MED25,ZNF653,GFM1,RAB9P1,C2,C4orf11,KRTAP10-2,TBC1D22B,APOA2,TCP11,PRDX2,FOSL1,ATP2B1,SLC9A6,FDX1,C6orf155,RBPSUH,PLOD1,CHRNA1,PCQAP,STX1A,DENND3,C21orf37,AXIN1,CDX2,SSX7,LYPD4,H2AFY2,PCDHAC1,APBA2,HMGB4,WDR74,CRBN,TCF7,PAPOLG,PAQR4,AKAP3,FCGR2B,PPP1R14B,AKAP1,TEX264,LEPRE1,CLDN14,CSF1R,NFAT5*
**GCM-72**	4,41E-01	14	*MRPL24,TAT,KIAA1826,IMPG1,C1GALT1,TCEAL4,SUSD2,GJA7,C6orf168,RPL18,NAPA,PRDM16,IL2,HAVCR1*
**GCM-73**	4,50E-01	26	*CYBASC3,DHH,C20orf6,RLF,LASS1,CPNE2,PPP2R5D,SLCO4C1,ASB17,XLF,POLDIP2,OSBPL6,RBM9,PMS2L1,NYD-SP26,PLA2G2E,TMC7,ZNF451,KCNK16,RBMY2EP,SULT4A1,RPS6,SYPL1,CAB39L,SERPINA12,PTPRC*
**GCM-74**	4,63E-01	131	*LPIN3,VPREB3,FAM83A,IGF2BP1,ATP5O,FAM13A1,KRT25B,FER1L3,RG9MTD2,AGRP,SPAST,FBP2,ITIH4,FCRLM1,SF3B1,RAB7,MYST1,MAP3K5,TPMT,TNFRSF13B,KIAA1644,PLA2G2D,PPWD1,C11orf11,ZBTB32,OLIG3,TOX,WAC,RAB11FIP5,JOSD3,HSZFP36,GNL1,ARHGAP22,TMEM54,GPR27,VANGL1,C11orf35,LSG1,ARHGAP25,RBBP7,LRRC8A,FLT3,CD19,MAP4K2,DUSP1,K6IRS4,SP4,ACTR6,MYCBP2,SPAG1,VCPIP1,EIF3S10,CACNB2,SACM1L,MOSPD2,OCIAD2,CR2,UBOX5,C5orf4,BAZ1A,PPP1R2P9,RPL7A,KIFC2,ZNF721,CARD8,SLC16A14,TDG,BAIAP3,FAIM3,CCR6,C16orf63,KLHL26,RND3,SYT3,GLMN,RASGRP3,FXYD6,AOF1,GVIN1,SUPT4H1,C9orf45,ARPM1,TLR10,NT5C2,LAMA1,DHX36,MAP3K7,SP110,FKBP9,NUP85,MALT1,FCRL3,BLOC1S2,CNTNAP2,TLK1,ANKRD12,PUM2,GTF3C1,HIST1H4C,FLI1,PYHIN1,C11orf34,LGI3,BLR1,DCTD,76P,FCRL1,RASA2,BLK,GLUD1,STRAP,S100Z,STAP2,AKT2,C1orf49,DAB2,TNFRSF7,ULK1,RCOR2,SIRT1,CRYM,C20orf94,RABGAP1L,LRRC61,NDUFAB1,SLCO1B1,ZNF140,NDUFV2,ZNF75A,NODAL,FKBP7*
**GCM-75**	4,65E-01	27	*SH2B,AP1S1,C22orf8,STX11,FAM3A,SNX10,CHMP5,VPS25,MAGEA3,SLC30A10,KRT2B,DGAT2L6,DKKL1,RAB11FIP1,TRIP4,SLC34A1,UBE3C,PSMB8,VMD2L1,FBXO44,NTRK1,ALKBH4,LACE1,CHCHD3,GAGE7,YTHDC2,DTYMK*
**GCM-76**	4,67E-01	15	*SLC1A2,HHLA3,LZTS1,TAS2R5,UBE2L6,ERCC4,CCRK,SLC35C2,HIST1H2BD,CDK9,CSNK1G2,FGF23,CTCFL,KRTAP13-2,PRG2*
**GCM-77**	4,68E-01	120	*KLHL24,NCF4,SERINC3,KIAA1274,MPST,ATP7B,BCMO1,ELAVL2,IRAK4,METT5D1,PAK1IP1,SURF1,RNU108,NIP7,OSTF1,DMRT3,NCAM1,DKC1,RNF149,OR51T1,ZNF545,GRB2,HOXD3,CD2BP2,CRYGB,MNDA,MERTK,SPRY2,EEF1E1,SSBP2,DNAJC7,SH3RF2,C5orf13,PCDH18,ANKRD50,CPEB3,DUSP12,BAIAP2L1,C17orf47,ZNF23,MGAT4A,OR5M9,DSCR2,NOL1,C2orf11,PSENEN,FADD,CTPS2,DKFZp686K1684,CLCNKB,ADAM28,CGI-115,PRLH,PREX1,BTBD7,RDX,CHST7,PUS1,CABLES1,BRD8,TANC1,PGEA1,CBFB,SYNCRIP,BAALC,CTBS,ACA33,GTPBP4,KIAA0020,PIGW,CMKLR1,VNN2,CASC3,HOXD8,SUPT7L,RPUSD2,GPR152,GCN5L2,PBK,DOK3,CLEC4D,*

			HIST1H2BI,TCEAL7,FPRL1,TBKBP1,KLHL11,MMP28,C19orf35,RFC5,SLC7A7,C16orf46,TCEAL6,SLC6A8,NPHP3,HIST1H1B,UBE3B,GRK6,C21orf25,SLC25A19,NUP98,RBM24,GTF2F1,ETV6,PAK6,MMACHC,SLC4A3,C12orf59,HMP19,LEPR,E2F4,LRRC27,DHX33,SERPINB1,NAPB,JTV1,ABCE1,TOMM40,CDKN3,ARPC2,RCOR1
**GCM-78**	4,78E-01	31	*IL9R,CTNNB1,ZNF440,PABPC3,RNF6,MASP1,C15orf28,BAP1,SNRPA,ZNF354B,RPLP2,MRPL2,EPS8L1,IFNA5,PRPF3,OGFOD2,PPP1R1B,AARSD1,AAMP,LTA,PHB2,AP3B2,TIGD5,RBM21,CASR,MAGED2,MRPL38,ST8SIA6,PURB,OR5H15,GMEB2*
**GCM-79**	4,83E-01	53	*LTBP1,XRCC1,GRIPAP1,LHFPL3,XCR1,KCNC3,MRFAP1L1,TACR1,PCGF5,OR4F21,LUC7L,TMEM23,C6orf61,PRPF18,COQ10B,TXNL4A,UBA52,TSG101,C21orf13,RTN1,CEL,KRTHA2,UBE1,FN1,EML3,SLC18A2,RENT1,HRB2,SOX5,DDX23,DCST1,OPA3,NXT1,SPATA12,ABCC5,B4GALT6,GRLF1,ZNF277,OR10K2,BECN1,TSEN54,URB,NBR1,TBCD,GP6,EVL,IGFALS,GOLT1B,IER3IP1,ATP6V1G1,FAM76A,DDT,AMFR*
**GCM-80**	4,92E-01	42	*ORC4L,JMJD2C,ZNF403,CLEC11A,PRDX5,DGCR14,SHOX,CES4,RASAL2,ACSM3,POLG2,PDLIM1,CXCL16,VMO1,SENP7,LAMP1,SOX30,ZMYND15,PRIMA1,MCFD2,TRPM4,MYO9B,RHOC,FAM50A,HNRPCL1,RG9MTD1,HSPC023,TFE3,CACNA1I,USP46,FAM113A,STAT6,GATAD2A,SLC22A18,FCHO2,MYOCD,MYL4,ALOX15,PLXND1,RWDD2,ZNF3,KIF1A*
**GCM-81**	5,00E-01	26	*SLC6A18,OR1S2,TP73L,GPR39,C14orf161,PRO0149,TH,KRTAP15-1,OR1L8,NOX3,C6orf113,TM4SF18,CDKL2,KLHL10,PPFIBP2,ZFPM1,NDUFB9,PARP4,NPAL1,GJB3,MRPS28,GLG1,RBMY1A1,OR7C1,OR1N2,ZNF257*
**GCM-82**	5,03E-01	27	*DEXI,PLAG1,ITGB1BP2,ENAM,C6orf128,RFX5,RDHE2,KCNC2,RASSF3,PSMB10,CD37,RP1L1,SPRR4,ASCL2,SMC4L1,FECH,TM4SF20,CRYGD,PDZRN4,SPIB,SYNGR2,ZBTB37,PAQR8,C14orf124,BCL11A,CHGA,PLEKHG5*
**GCM-83**	5,12E-01	20	*MAGEA2,HCN2,LMO3,C18orf25,RPL12,SCN7A,PDZK1IP1,P2RX2,WDR49,FLG,C7orf25,PCSK5,CDK5R2,RYR2,SCGB3A2,TRPV4,UNQ9356,MPN2,ANKZF1,CD1E*
**GCM-84**	5,14E-01	18	*TDRD9,GPR172B,FAM71B,EPC2,CREBBP,TCEB1,LSM16,MSX2P,APOH,SDHB,MYST3,C7orf11,LCE1E,PES1,LYPLAL1,ABCA12,GALM,TUBB*
**GCM-85**	5,14E-01	30	*NOVA2,RNU40,SNRPD2,C13orf18,OR8G1,IFI44,C6orf136,CLCN6,SYNE2,HSPBAP1,G1P2,PLOD2,DHDH,HERC6,ZNF213,IFI44L,PSCD1,SPRED2,HTR1B,C11orf36,ZNF526,HOM-TES-103,SLC22A7,LY6E,CLSTN1,SREBF1,BIRC4BP,HYAL4,ZNF365,OR51F1*
**GCM-86**	5,17E-01	23	*SUZ12P,NDUFA12,C9orf93,DEFB126,PCDHGA9,GNG4,ASAH3,SCRL,KRTAP21-2,CLYBL,KRT7,MYO6,MS4A3,SNX26,CSPG3,GRIN1,GABRA1,CD300E,PPOX,CCRL1,ST7OT4,TIGD6,UNC45A*
**GCM-87**	5,17E-01	48	*PLEKHN1,C15orf43,TTC1,SLC45A2,THAP11,HP1BP3,TM9SF1,TMEM111,THSD4,CLOCK,AQP4,NSUN3,PRDM14,HAO1,CHRNG,CWF19L1,N4BP1,KCNK15,KIAA0971,BARD1,P2RY5,PKNOX2,C10orf118,TMEM32,ZNF26,KLHDC8B,EZH1,C6orf122,CDC37,C8orf55,TACR2,ZNF567,MGLL,RHAG,PRDM10,MAN2A2,DUSP11,FBXW7,IL11,C6orf94,C9orf23,SLC17A4,ADCY3,FRMPD1,CYP3A4,C14orf142,OR52H1*
**GCM-88**	5,17E-01	40	*TENC1,STOML3,C7orf20,RNF175,AMHR2,C14orf168,ITPKC,OIT3,ZIC2,OR52R1,AMIGO2,WDR6,C9orf153,CNIH4,RNU54,ANGPTL4,ARL17P1,NFKBIA,COX6A2,PHLPPL,OTX1,C1orf127,OR3A3,DLX1,HAS3,LCMT1,TMPRSS11A,C6orf206,ZBTB16,TSSK6,UBXD7,PSG7,HSPD1,KRT1B,SPACA1,GOLGA8G,PHACTR4,SPTB,DEC1,IBRDC2*
**GCM-89**	5,21E-01	18	*POLR3C,GALNTL5,DHRS4,AHSG,NIN,SLC5A9,A4GALT,ROPN1L,CFH,TIGD2,DDX3X,PDGFRA,NCAM2,DHFR,C1orf170,TMEM108,TFPI,KIF5A*
**GCM-90**	5,22E-01	31	*ARHGAP6,FAM78B,EDN3,PTPRN,KLHDC5,TSGA10,C1orf120,DDX27,TMEM70,ZAP70,RNASEN,RPL18A,SLC22A4,AHSA2,ODF1,TREM2,CLEC7A,GLRA3,HRBL,AADACL1,MCART6,STARD3NL,GNGT1,NT5C1B,DUS4L,C1orf121,C20orf35,SMAD4,KRT6IRS,MAGEA10,PPIA*
**GCM-91**	5,44E-01	23	*LGI1,DDX25,NPAS3,EXOSC10,TXNDC6,TMPRSS5,RBMY2FP,PLEC1,CTSC,HIST1H1T,DEFB1,HAS1,SLC43A3,CHRNA4,C20orf152,OR4D9,SH2D5,POU3F4,IQCC,FANCM,VGLL1,TPTE2,KIFC1*
**GCM-92**	5,46E-01	27	*DIAPH3,SNX30,CLDN20,PUS7,TMEM102,C16orf44,IFT122,MYL1,NT5C3L,SLCO6A1,GDAP1L1,TIMM10,C12orf36,CLEC2D,ZNF268,LAT,PIG38,HMGCS2,PNRC1,NAGLU,MRPL45,IFITM1,FAM106B,C6orf143,OR52A1,KCNT2,C1orf87*
**GCM-93**	5,64E-01	16	*OVOL2,OR5BU1,POLR3A,GOLGA4,NXF4,NANOS3,GIPR,OR5A2,NFATC3,UBL7,APEX1,MUC6,SLC2A4RG,HFE2,PKD2L1,KCNA5*
**GCM-94**	5,82E-01	74	*RAB18,CORO7,RNF135,PLD4,DNMT3B,SLC26A9,NRL,CC2D1A,SNAPC4,AASDHPPT,NBEAL1,C7orf27,SIDT2,ZMYND17,SI,IKBKB,KIAA1754L,KIAA0372,ABCB8,C6orf97,PTDSR,TMEM39B,RASA1,RPA4,ENPP4,SLC22A17,SLC12A5,GABPA,TAF15,SSFA2,C6orf151,ANKS6,OR5M1,EN2,CAGE1,STAB1,ZFAND2A,NUFIP2,OAZ2,USP33,C20orf43,HTRA2,C8orf1,KRTAP19-4,EIF3S7,PSMA1,USP50,ARL11,PIK4CB,C1orf65,IRF2BP2,OR10A3,SNF1LK,ETAA16,TMEM103,LRRC20,DSCR5,USF2,PELI1,DMAP1,SARS2,RNPEPL1,ATP6V1E2,C17orf67,TCHHL1,NARG1L,AMBP,CCNB3,CASP7,SLC2A4,ARFGAP1,CABP4,POLN,ZMYM5*
**GCM-95**	5,89E-01	74	*C21orf6,ERBB2IP,C12orf41,TAAR1,USH2A,RBBP4,KLK10,PAK7,GNAT3,DTWD1,FGFR1OP2,SCN3B,PCDHB6,PAX6,GADD45A,ICAM2,EPHA8,CXCL3,EHD1,FFAR1,PRMT2,OR6C76,ABCA13,OR4N2,DNAJC8,MEF2D,DSCR10,COL12A1,GMFB,H2AFB3,USP2,BIRC1,F25965,NME1,CAPSL,SCN10A,CST11,TMLHE,VTCN1,SMR3B,GLDN,7A5,PCDHGC5,DUSP3,RABL2A,EIF3S3,ACOT12,RTN4RL2,OR5H6,LARP4,OR2T10,CACNG7,CSNK1A1L,KRTAP8-1,ALKBH5,SMURF1,NLE1,LOC388692,TDGF1,FMO5,DSCAM,NR5A1,CDC42EP3,QTRTD1,MAPK8,LPIN2,PRB4,ZNF79,NBR2,C5orf3,SAMD14,ZNF383,RBBP8,OR2K2*
**GCM-96**	5,97E-01	30	*PRMT7,DUPD1,NRAP,MBD6,MORC3,LOC145845,C3orf33,LSP1,DNAJC5G,A2M,CDKAL1,SRP14,HPS3,PFDN5,CMTM8,MIB1,WFIKKN1,ANKRD37,KIAA1219,CD99L2,SP1,TAF1A,SCAP,SERPINF1,SLC2A14,ZNF189,CACNA2D3,EXO*

			C8,MTRF1L,DIO2
**GCM-97**	5,99E-01	15	*SLC1A6,C1orf36,LYK5,C17orf78,CDC2L1,OR4D6,CDH17,MRPS26,HIST1H1E,TADA3L,DHRS8,PEX5L,UPK3A,TTC16,GABRG1*
**GCM-98**	6,06E-01	26	*RGS13,NID2,KLK8,GSTA5,MRPL48,DERPC,PSMB7,C17orf64,ZNF155,KIR3DL3,ITGA2,OR7G3,OR5V1,CDR1,ACF,PKD1L3,TAS2R44,C3orf58,GPR139,TMEM40,NMT1,MRGPRD,OR10H2,PRG-3,VMAC,SMCP*
**GCM-99**	6,11E-01	19	*UGT1A4,OR4P4,BSDC1,PCLKC,RPIB9,HS747E2A,BAI3,TNFRSF19L,SYN1,TAGLN,PRM1,TRIM33,RNF41,SLITRK5,TPM3,USP32,PLD5,BID,CORO2A*
**GCM-100**	6,13E-01	23	*ESRRG,COX15,SLC10A1,CYP26C1,CLDN2,FIBCD1,BRDT,ANP32E,MYT1L,TNRC6B,C9orf110,WFDC10B,DHX8,HNRNPG-T,IGSF4D,HIST1H3B,ZFP95,CYP24A1,PLXNA1,BRP44L,AUH,TGM7,TRPS1*
**GCM-101**	6,26E-01	24	*P2RY1,KCNK13,ST8SIA4,HLA-DMB,CEACAM20,ZIC5,IAPP,KCNH7,UBXD3,CNNM2,CIITA,UCKL1,H2BFM,C1orf52,ADAMTS20,B3GNT6,RTDR1,MCOLN2,ERN2,KLHL15,KMO,GRM2,CGI-09,C13orf12*
**GCM-102**	6,36E-01	20	*CKS1B,RAPH1,SFRS16,LUC7L2,KRTAP9-5,PFKL,TNS3,OR52B4,PPM1B,TRAPPC3,LCE1B,ZNRF1,OSTalpha,POU3F2,PVALB,MAFG,OR2M2,RPL9,PRP2,PPP2R4*
**GCM-103**	6,39E-01	251	*LRRK1,ASL,C1GALT1C1,CREB3L2,GALE,C6orf118,MRPL1,EPHB2,GSPT2,C8orf58,TINAG,GMDS,CHI3L2,ARL6,KCNH5,CYP27A1,KCNN3,CFHR2,YPEL5,LGALS1,SCNN1B,TMEM84,PPARD,AFP,MTO1,FIGN,DEK,RNU31,XRN2,ZNF624,SFRS5,C21orf42,C20orf71,LCE1F,RPRM,MRPS10,TRIB2,TAS2R4,ZNF682,SEPHS1,HSD17B12,EIF2B1,HUNK,ZCCHC2,GTPBP1,PCOTH,GNE,NRP1,ARPP-19,SIPA1L3,UNG,RIC3,BTBD4,TUBGCP3,BTN2A1,LRRC7,DCC,TBPL1,TMEM104,SULF1,COL9A2,PRKRA,ARPP-21,ZNF527,CD40,MC4R,TMEM59,ALKBH3,TCF20,ZF,ARL5,APOL2,LRRC28,THEM2,KLHDC7B,KIF23,PPAPDC2,GPR149,RFP2OS,ELMO1,CEP63,RNF44,SRPX2,FABP5,HS3ST5,POLR3F,EPPB9,PLA2R1,KIAA1086,ATPIF1,CARS,PARVG,RTBDN,SLC1A4,ZNF406,RPS6KL1,DDX52,FCRL2,C1orf108,NDEL1,SUHW1,FBXO3,FKBP1A,ITGA1,FEZ1,DDX18,RAF1,ZNF499,UGT2A3,SHANK1,WBSCR22,EFCAB1,ACBD7,ARV1,JUP,TMOD4,ZBTB1,PROCA1,TNNI2,MKKS,RAP80,PEX11B,FZD5,WDR61,C11orf16,KCNH3,RAPGEF6,MVK,PLEKHG3,PLGLA1,ESPN,POU1F1,CNDP1,IGFBP3,PPHLN1,TETRAN,TESK1,OR2T1,APOM,CRSP3,PRPF4B,RKHD1,ART5,TTTY5,G6PD,PPP1CC,FAM3D,CHST2,OR6T1,C14orf115,HGF,HOXC5,DOPEY1,TAF11,MPHOSPH10,TPT1,ASTE1,SUV420H1,RPS11,OVCH1,DSPG3,CCDC71,PDHB,C4orf6,HOXB9,LENG4,TTBK2,CBFA2T3,SLC16A7,TES,KRT8,ATP13A4,GNG7,HOXA10,FAM62B,LOR,C21orf94,ASPHD1,CYP2E1,MYH16,ATOX1,ACTN1,AP1G1,ST8SIA5,THAP7,KIAA2013,HARSL,KIAA1212,TMEM86B,TMEM87A,SMYD3,RPL36AL,ZNF690,NECAP2,RFXAP,TLN1,WDR8,NR1I2,ATG16L2,PITX2,C8ORFK36,PTPN6,CIT,TRAF2,SAA1,TMEM9,MPP3,EPN3,NEU2,RARSL,DAZAP2,CDCA1,GCNT1,DCBLD1,HDAC9,OPTC,MAGEH1,GNG3,ARL1,MMP20,CMAH,POLR1C,DERA,LOXHD1,TP53RK,C17orf75,LRRC52,UNQ501,NEB,C20orf195,SOD3,EFHC1,ZNF35,SRP46,OGDH,REG1A,SLC22A1,SOX14,STARD4,OR51S1,CABLES2,OR5AK2,C17orf44,TWISTNB,TAS2R10,LHX6,OR52E8,EEF1A2,COL15A1,TNIP1,MARCH5*
**GCM-104**	6,40E-01	38	*RSPO1,GPC1,GGTLA1,C1orf83,VIPR2,C3orf23,XAB2,TMEM129,ZHX1,NEK6,CSAG1,OR52I1,OR2T8,TBC1D13,ADAMTS9,HS3ST6,OR5A1,UCP2,CD226,TTLL10,COX7A2L,SH3BP5,KRTAP6-2,OR2S2,CRADD,CCDC17,APBB1,TRIM7,WHDC1L1,ZNF679,CHKA,FLJ37201,PALM2,C7orf23,MARCH2,KLRC3,USP19,SIAH1*
**GCM-105**	6,44E-01	62	*CD44,MEFV,ERCC3,ZBED3,IGFBP2,CASP8,FMO4,APPBP2,TRIM4,BCDIN3,SLAMF8,YSK4,RANBP10,SPRY3,C7,CA2,GALNT13,ATP6AP2,ALX4,PLCB1,ZSWIM5,HAGHL,DNAJB6,PTGDS,FMO6,APBA2BP,EBI2,GPBAR1,ZNF76,MPPE1,ALB,INS,HYPB,NAP1L2,RGS1,FBLN5,CNTN1,KRTAP11-1,PKIG,HSN2,PLA2G4E,DEAF1,TAF1C,BHLHB3,GUK1,FAS,TCEA2,RPESP,SLC35E3,PRKAB1,SIGLEC11,BMX,PFN3,SERPINB12,C10orf81,PTEN,PPFIA2,ARL8B,CAPN3,GBP3,ZIC1,FUT4*
**GCM-106**	6,52E-01	18	*GADD45GIP1,F8,REG1B,VENTXP7,RNF190,C9orf128,P2RY2,RBM35B,GFRA4,PROL1,FXN,AMOTL2,KRT25D,KIF27,IFIT5,OR1A2,TFF2,C11orf42*
**GCM-107**	6,69E-01	17	*ZNF694,SREBF2,PAK1,NUDT22,ZYG11A,DDX42,ABHD14A,SCNN1D,SCPEP1,K5B,RPS18,ANKDD1A,RPS29,CXCL12,IBRDC3,GGT1,WDR42A*
**GCM-108**	6,72E-01	19	*HAND1,AQP10,TXK,XYLT2,ECE2,GPR62,FBXL12,C20orf141,SNAPC1,EREG,PRKAB2,EPGN,TAS1R2,ONECUT2,SOX21,TBC1D5,ZNF615,BAHD1,MRPS33*
**GCM-109**	7,05E-01	35	*DUS3L,ATP4A,TLX2,FGF5,MSMB,CLLU1,SLC5A4,CLDN17,ZNF184,OR10AG1,RPH3A,IGF2AS,DGKI,KCNG3,NUP153,B3GALT3,PRKACB,FAM19A4,WDR5B,AGC1,CLCN5,PRPF19,OR5R1,RNF7,OR4C12,CYP2C9,NFE2L2,HTR1A,ZNF595,RNF8,TP53,PET112L,IL17,DDB1,NBN*
**GCM-110**	7,23E-01	42	*CDGAP,NALP8,TEDDM1,EEF1B2,CRX,NUDT14,RHBG,USH1G,UBE1L,FRMD4A,THUMPD3,B4GALT4,UTS2R,MAML3,RELB,C1orf159,LMO6,PTK2B,IGBP1,VAMP4,ST7,OR10A7,ZCCHC8,NR1H3,KRTAP5-6,TBC1D1,MFHAS1,PRKCZ,ACBD3,ARID2,FAM9B,ARCN1,KIAA1008,ZNF628,TBX5,C9orf71,GOLPH3L,FLNA,MGC16025,ASCC1,ZBTB17,SLC15A3*
**GCM-111**	7,27E-01	30	*SLC14A2,RHOT2,C3orf57,UBB,C21orf127,MON1A,DPP9,ZNF282,SPO11,MCM7,RNF128,CART1,C1orf19,ICHTHYIN,U2AF1,BAT2,VENTX,APOL6,FMNL3,HCG4P6,KALRN,SYCP3,KIAA0913,PRDX3,RBM35A,MTMR12,GSTM4,OR52M1,RAD18,C3orf27*
**GCM-112**	7,31E-01	19	*TP53I13,PROSC,USP38,C11orf46,ARSK,TRPC4AP,CFL2,OR51A4,STRN4,QKI,PCMT1,TPD52L2,KCNIP2,C14orf125,KIAA1967,TMEM52,SP3,PTPN18,NUCB1*
**GCM-113**	7,32E-01	18	*CNIH2,OR2T4,ZMYND11,TNFSF15,MYBPH,OR1S1,IL19,OAZ1,OR4D5,FOXQ1,WDHD1,SPAG9,RNPC2,FAM71A,ZNF469,LTA4H,SEC10L1,ADAMTSL5*
**GCM-114**	7,41E-01	31	*RAD50,FAM77D,GDF15,WWP2,CHEK2,RARRES3,TGFBR1,AKT3,C18orf16,ILF3,ESCO2,PRM3,BBS7,PAK4,STX18,C20orf132,CRELD1,KLK11,U2AF1L3,TD*

			RD7,ZNF442,NYD-SP11,ZNF304,FCHSD1,C13orf24,FSHB,ZNF645,USP15,OR1G1,NAB2,IL26
**GCM-115**	7,63E-01	112	*TMEM16A,COL6A3,COL3A1,CYP2W1,TMCO2,OR2Y1,EDNRA,HES5,OLFM3,ZNHIT3,PDE9A,ACTRT1,BACE2,COL5A2,APXL,CA5B,C12orf43,CTSK,EPHB1,KCNN2,C20orf96,RKHD3,CTNNBL1,TNFRSF10B,CTAGE5,CA11,KCNJ13,EPHA10,SORCS2,BHLHB5,PLA2G12B,ATP6V0A4,SPATA3,SOX6,SLC9A5,DIP2B,IL3RA,MICAL-L2,KIF21B,PSMAL,GNAS,C6orf130,CDH13,C14orf150,PPIL4,PAXIP1,VMP,CHST6,KDELC1,MMP11,HIVEP1,COL5A1,CHST1,MTMR11,SBSN,MEIS1,RFC1,ZNF31,RFFL,C14orf37,ASPN,DAZL,PPEF1,CPT1C,SCRIB,MUC5B,C20orf177,APOL1,MAGI1,IDH3B,PRAMEF8,ALAS2,ATP1B4,MXRA5,KIAA1324L,SLC12A1,IL29,STATIP1,WWC3,MYOM3,LRRN6A,COL6A1,COL1A1,CEACAM16,KCNK2,MAP2,SLC25A4,GPR162,GEMIN4,BMS1L,RGL1,PIGY,ARSG,POSTN,SEMA5B,C20orf22,THY1,MRPS18A,ZDHHC9,MEIS3,C1QTNF6,LOXL1,TYRP1,ADAMTS12,ATP8B1,TRIM6-TRIM34,OR5AU1,OR10G3,GOSR2,ATP6V1B1,CLEC4C,TBX22*
**GCM-116**	7,75E-01	28	*CABP1,SERPINA11,GATM,ELOVL2,CHN1,IL22RA1,OR4C16,BPIL2,GLIS2,HTR1E,OR1L3,C14orf73,SYT13,HIST1H2BA,KIR2DL3,IGSF2,ING4,BMP3,DCTN1,LOC399715,FOXF1,OCLN,ANG,CGB2,TAP2,PTER,LRRC17,ARL9*
**GCM-117**	7,78E-01	24	*LIM2,TMPRSS4,GATA6,ATP6V1G3,NOTCH2NL,C14orf21,OR52L1,KCNIP1,PERQ1,MMP25,GPX5,MED19,ADPRHL1,TSC22D3,ERCC1,USP29,IFT20,CLSTN3,OR1A1,KIAA0141,SCOTIN,FKBP6,LYPD2,ARRB2*
**GCM-118**	7,89E-01	18	*WRB,BCL2L14,TOP2B,BCAP29,C1orf63,MIB2,YTHDC1,FAM11B,SCAMP3,TSPYL1,PITPNA,LRRFIP2,MITF,S100PBP,ZNF594,NANP,RPS8,MBNL3*
**GCM-119**	7,89E-01	22	*FOXR1,SENP6,AIP,IFNA16,HILS1,MOBKL2B,OPN5,C9orf98,INADL,TREH,HAO2,mimitin,CTBP2,SLCO1A2,CMTM3,C14orf70,IL20RA,TAS1R1,ARL5C,HSPA1A,ZNF287,ZNF302*
**GCM-120**	8,03E-01	55	*CAPN12,GlyBP,NPAS2,GNAI3,POLE4,TMEM16B,LOC541473,HSP90AB1,COPA,PBX1,OR52E6,PITX3,CASKIN1,SEC24D,BCL2L1,MTMR8,ZNF432,GJB7,PABPC4,C8orf4,OR9G1,ILK,PELI3,KIAA1751,ZNF691,COG6,LOH12CR1,HDAC11,GCSH,TUBB2C,RBAK,TCF15,COL11A1,TJP2,OR4A47,NDRG4,ATCAY,ESPNP,GRM1,SFMBT2,C1orf179,KBTBD3,NAV2,HIST1H1D,BTBD8,NPNT,PCBD1,INGX,KNDC1,MRLC2,FABP2,NTHL1,OR7G1,RNUT1,CD200*
**GCM-121**	8,09E-01	17	*PVR,MYO7A,AAAS,TLE6,MAP3K3,SEZ6L2,DYRK2,GALNS,ZNF185,KLF7,C6orf134,TBC1D10A,GGN,UCP3,ASB6,PPA2,SCRN1*
**GCM-122**	8,24E-01	33	*KIAA0514,MAK10,ZSWIM1,USP8,MAP1LC3C,CLDND1,PRPF31,BAT2D1,UQCR,LY6K,ANKRD11,SMG6,CCDC49,GSTP1,C20orf26,PTPRJ,FCHSD2,GYS2,C6orf71,CD63,STAU,SMPX,EPIM,NUDT16,ZNF205,DHX15,CYP2J2,TDRKH,CXorf42,MRPL43,ITR,SPACA4,DHX29*
**GCM-123**	8,33E-01	30	*EML1,PHF10,C6orf27,TGFB2,AACS,KRT20,THH,TRSPAP1,IFNA14,TMEM85,PRDM2,RHOBTB1,OR5K2,HLA-DPB1,MTFR1,OPLAH,CACNA1E,RHO,SH3TC2,C1orf32,HEXIM1,DOCK11,HSCARG,GUCA1C,ATP8B2,HSPC152,CYP17A1,ZNF696,UST,CST8*
**GCM-124**	8,43E-01	16	*RBPSUHL,NPHS2,FADS6,SPPL2B,TOP1MT,ABCB7,SLC39A2,NRXN1,PDHA2,TMPRSS11F,BRCC2,HSD17B4,TRPM5,KCNC4,PADI6,KIAA1840*
**GCM-125**	8,53E-01	16	*RTN2,EPS8L2,DKFZp686O1327,FOLH1,SURF2,PCDH7,LZTR2,IREB2,ITGB1BP3,NUDT12,LRRTM4,MCHR2,UNQ2446,RORA,IL1F5,SLC7A13*
**GCM-126**	8,76E-01	30	*RBM7,C20orf29,PCNXL3,GPR56,C12orf23,C18orf51,HECA,CREB3L1,GALNT1,C3orf62,NADK,NTSR2,PDE4D,JUB,RELA,SMAP,MAP3K7IP2,TPST2,ZNF250,CLIC4,ZNF160,PAFAH2,C10orf128,C1orf141,POFUT1,INPP5F,TFEB,ZFYVE9,ZNF295,OR2M5*
**GCM-127**	8,79E-01	15	*DSPP,TCF2,RUNX3,ASB15,CSPG6,FBXW2,KIAA1333,SLC6A7,CLCN3,GPA33,MLN,TOR1AIP2,CDH22,MME,HCN4*
**GCM-128**	8,94E-01	22	*ZC3HC1,RAP2C,IGSF6,ZNF93,PIK3R4,OSBPL8,FAM5B,BTRC,NAPE-PLD,THTPA,C6orf154,CLC,XAGE5,PDIA5,HSPBP1,C4orf16,C20orf144,POU6F2,SLC38A6,L3MBTL,C3orf28,CAPG*
**GCM-129**	8,95E-01	26	*TBX6,NOM1,SH3BGR,FREM2,FAM55D,DKK1,ARL14,C21orf45,KIR3DL1,SIRT6,GRAMD1C,MS4A10,WNT10B,C1orf125,LCE2A,TDO2,MYOG,KIRREL3,LGALS8,OR2B3,TUBB8,AKR1CL2,PCNA,NKD2,GAGE6,ATP10B*
**GCM-130**	8,97E-01	27	*GIF,OR52W1,MOBKL2C,C1orf186,KLK3,CRSP7,SCD5,EXOSC4,MRPL52,PRRG4,EXOSC1,CDH24,SURF6,GPX7,ATP11C,TXNDC2,ZNF137,PPP3R1,ZNF354C,PRR10,CYHR1,SLC31A1,ZNF585A,UNC13A,ZNF512,KIAA0528,GRPEL1*
**GCM-131**	9,02E-01	23	*RNASEH1,LRRC29,EIF2S1,TPRX1,ME3,FAM43A,CACNG5,SEZ6L,CLTC,FOXP1,RBM13,SAS10,FXR1,TIGD1,LEPREL1,VDR,RBM3,MATN1,C9orf7,KIAA1815,MTFMT,CPAMD8,KRTAP2-2*
**GCM-132**	9,09E-01	22	*FBS1,GMPR,BAG2,THRAP2,MAT1A,FAM20A,APOC3,MPI,OR8B3,DENND1A,HRAS,PPY,ANKHD1,KLF17,ZNFX1,NUBP1,IHH,SLC6A15,PRDM13,BMP1,OBSL1,TCEB3B*
**GCM-133**	9,13E-01	19	*GLT6D1,PCDH20,ENTPD8,TTBK1,TNFRSF4,DLG1,ALDOA,KSR2,CACNB4,RGR,BTBD2,RXRG,SYN2,C20orf52,SCO1,C17orf41,ODF2L,FAM12B,MATN4*
**GCM-134**	9,18E-01	17	*CMTM2,CSTF1,LAMA3,PADI4,S100P,INVS,SRY,ZNRF2,OTUD7,ZDHHC22,IL8RA,FGD2,GPR173,POPDC2,TPCN2,MUC13,VNN3*
**GCM-135**	9,25E-01	37	*BAI1,ZNF335,PCNT2,LRRC51,CCL20,C15orf21,TTC19,PARP16,SCFD1,DEFA5,GPD2,ANGPTL3,UBE4B,BMP2,HSD11B1,TNFRSF9,RAP1A,PAK2,FANCA,C4orf8,C9orf46,C15orf48,SH3MD4,RASGRF1,DNASE1L3,LZTR1,TFEC,MAP2K3,ABCC12,LGTN,SNAP91,CTAG2,EEF2,GUCY1B2,MAP3K4,EIF3S6IP,ACBD5*
**GCM-136**	9,33E-01	225	*UBE2D3,VGLL4,C3orf21,RALBP1,UBE3A,OXA1L,C17orf70,LSM10,ALKBH8,TMEM1,NUP35,PRDM7,LYN,C20orf98,EMILIN1,SALL1,BLM,LHX4,CCNT1,CYSLTR1,KLHDC3,COBRA1,MYH6,NLF1,PIK3CG,APXL2,C19orf4,SAT,TBX1,LEMD3,C8orf38,ELMOD1,PTGFRN,CNTD,MLLT7,RASL12,VPS37D,DDX20,OR52K1,CEP350,ZNF297B,PADI2,ARMC1,NKRF,NT5M,PTPLAD1,GUSBL1,C1orf35,PLXNB2,ACD,KIAA0195,DTNA,PLCB3,C8orf53,PLEKHF1,TFIP11,SGEF,KCND3,COL4A3,DNAJB5,STK38L,NES,CDC2L6,HLA-E,ALG14,RNF19,ZNF576,SRPK2,IFNAR2,TTC17,SLC23A3,SLC35D2,FAM99A,BTBD14B,ZNF573,FREQ,NRBP1,ASH1L,COPG2,CDC42EP5,ZNF364,SC5DL,*

			SAMD1,C14orf104,PHC1,UBE2L3,WDTC1,TUBB4,TMEM5,NDE1,EIF5,DYRK1B,C3orf9,SLC9A1,FAM98A,DNAJB14,TXNDC9,ARMC8,TSR1,ADRA1B,PSG8,FLJ46906,TM7SF3,C1orf82,KLP1,SLC3A1,SPATA8,TMEM30A,TBRG4,TULP3,PRKAR2A,DNAJB2,CCAR1,EP300,PLA2G3,ZNF281,YIPF6,DIP13B,PAPD5,KATNAL1,CATSPER3,UROD,YPEL4,SLC25A5,ST3GAL4,C13orf23,ADNP,SLC2A11,RPS6KA6,RBM4,YPEL2,FSCN1,CABIN1,SRP19,UBE2N,IFIT3,TAX1BP1,SCDR10,E2F6,ARHGEF16,ITPR2,YWHAB,NOX1,SUZ12,OR5BF1,SF3B3,SDCCAG1,MUM1,CEP76,SETD6,NOL5A,FLG2,UBE2O,DLEU1,GLCCI1,LRFN3,LMO4,C9orf112,PLSCR1,HEL308,COL4A3BP,TAF6,ASXL2,ATG9A,DUSP18,ABHD4,C15orf15,NFX1,CDV3,CEBPZ,LTV1,GTF2B,MRPL20,PDGFB,ATP6V0A2,YY1,BCOR,C6orf66,ARMCX1,TAZ,CCNI,TNPO1,PIAS1,UGCGL1,WDR12,MLANA,EIF2C2,USP35,ABCD1,ZNF157,KIAA0133,ASS,C20orf149,CAMK2G,TCAP,PTPN21,ZBTB40,CHCHD2,CNIH3,RNU73,CDK10,CARD4,PSPC1,SLC7A1,TTC15,FZD9,MRPL50,FBXL7,IL18BP,HEBP1,MYO1E,ZCSL3,PLAC4,PPARBP,LRRC6,HYPE,TFB2M,FLCN,ZNF609,ING3,IL1F10,OR11G2,FAM55C,DRB1,UNQ1912
**GCM-137**	9,36E-01	29	*OR2AG2,XIST,CA6,ZFY,BCL11B,CHAT,PRKY,UTY,USP9Y,TRIM16,TTTY15,CENPC1,OTUD3,NAG6,SMCY,FGF1,LRSAM1,C19orf21,NLGN4Y,VGLL2,EIF1AY,CHCHD8,CYorf15A,RPS4Y1,C9orf57,OR2L13,CYorf15B,OR52J3,FGF12*
**GCM-138**	9,36E-01	20	*RYR3,RNF12,OR6X1,TNFRSF11A,TMEM128,DPP6,H1T2,PCDHGC3,TAAR9,FGA,ZNF407,C20orf79,SLC22A12,P18SRP,HISPPD2A,OR2F2,BCAN,FBXO24,MIP,CLEC9A*
**GCM-139**	9,42E-01	26	*NRXN3,HIST1H4F,MB,SPATA4,NT5DC3,HM13,DUSP4,BZW1,ZNF627,NPY,SDC4P,C1orf146,HOXD13,BICC1,TMEM74,HOXA1,SESN2,OTUB1,CTNNA3,C1QL2,FXYD7,DBI,HTR3E,RBX1,HTR7,LTB*
**GCM-140**	9,48E-01	69	*ADI1,DCI,AHRR,NDRG1,SLC25A24,DP58,SNFT,PGLYRP1,SKI,UTP14C,OTUB2,KRTAP10-7,CGGBP1,MKRN2,CTTNBP2NL,ANXA2,ALDOC,TBP,ACYP2,SERINC2,ZNF11B,ZNF616,ARL6IP4,PSPN,ADRB1,ADORA2B,FCER2,RNF25,MFN1,TPST1,NAGS,PPP2R2B,MRPL42P5,ADAMTS2,YEATS2,KIAA0409,CXorf34,DDIT4L,IFNA7,QSCN6,C6orf59,AKAP13,HEXDC,HECW1,HBS1L,KCNE4,ABCC1,SLC25A3,XPO4,CP110,ANAPC5,PABPC1,RAB40B,VPS52,C10orf35,HCFC2,MED6,BRUNOL5,FBXO47,AGMAT,RPL4,FABP1,CORO1C,MAP3K1,BDP1,ZNF700,ZZZ3,WDR70,UTP15*
**GCM-141**	9,54E-01	34	*F7,CYP4F2,MFI2,CCDC69,IK,DBX1,HHAT,AMAC1L2,CSDC2,ELK3,MBD3,C1orf88,IL20,SLC24A5,OR51A2,STAC3,MAPK8IP3,MAS1,CD276,MAFA,MTM1,PTPDC1,GPR157,PARC,C14orf111,IRGQ,NR4A3,ARG1,WDR47,RAXLX,DHX38,SETDB1,ANKRD28,BRF1*
**GCM-142**	9,55E-01	56	*TLE1,MOV10L1,GTF2A2,PCDHB17,TMCC3,RCL1,SKP1A,IL10RB,SYT14,C1orf60,CHDH,CD84,HDAC6,COLEC11,ASTN2,C12orf31,C15orf37,GATA4,CASC2,PDLIM5,ZNF256,PRND,VARS,NARG2,OR8G5,MAGED1,SLC43A1,PLXNB3,GAS2L3,PACSIN3,TMEM44,BNIPL,SLC22A3,DSCR3,EGFL8,ALS2CR12,SLC30A8,HMBOX1,RGS3,EPX,GPM6B,CDH10,BMP2K,C10orf119,XAGE3,TM6SF2,PDILT,CAMK2D,LRRC42,KIAA1914,ARPC5,RDH12,RET,OLR1,CHST5,ATOH8*
**GCM-143**	9,61E-01	30	*RNF141,IL1RAP,RBP2,PKD1L1,DDX10,UNQ6975,GART,GRM7,C18orf26,FLOT2,CDC40,FAM11A,KIAA1838,SIGIRR,TDH,MYBPC1,DFFA,MYADML,EXOC1,RNASE8,PCSK2,TFAP4,SLC7A14,HRH4,SF3A1,BAI2,FAM20C,FBP1,CEBPB,FMNL2*
**GCM-144**	9,79E-01	82	*GLRX5,GMPR2,RAB19B,OR10H3,PCM1,NR6A1,MAPK11,Tenr,CRI2,C6orf25,C21orf7,SCN2A2,PRSSL1,RNU64,HIST3H2A,TPR,ZNF558,HS3ST4,ZPBP2,ERH,PMCHL1,GPATC4,A2BP1,MOCS2,KIAA0090,OR51I2,C9orf21,LSM8,C2orf18,RSHL2,PHEX,PCK2,SYT7,PCSK6,PPAP2C,TH1L,C21orf59,TCN2,SLC26A8,RPP38,SCT,KCNK4,TMEM76,SYNPO2L,PIGH,COG8,SS18L1,GPR6,PRMT3,C1orf162,USP39,THRAP6,ANKRD30A,NDUFA10,FOXN1,KBTBD2,OR5K1,IPPK,STK32A,ST3GAL1,C21orf121,MYOD1,GPR24,STMN1,RAD52B,EIF2AK1,TSPAN1,NIPBL,RNU35B,IL31,PLRG1,SYCP1,PANK1,VLDLR,FBXO38,H19,HIGD1A,OR13D1,PNLIP,MYEF2,PCDHA12,RNF13*
**GCM-145**	9,83E-01	28	*RFPL2,MIR16,NUDT6,UTS2D,LHFPL1,ZFYVE27,P2RY6,ICOSLG,DGKK,AKAP5,USP1,ZNF482,SAMD12,LIPF,SLC5A1,TBX21,C10orf47,OR2L2,LBX2,SLC25A32,SRrp35,KRTHB5,BZRPL1,OR10G8,RNF186,CPEB2,DUSP22,TTTY18*
**GCM-146**	9,85E-01	28	*MAP1B,PTPN2,POLR2H,APOBEC3A,CXorf17,C14orf105,psiTPTE22,ABL1,MCMDC1,LECT1,BMP15,UROS,NUDT19,CEBPD,PPP1R1C,C10orf62,G1P3,IRF3,IL6ST,RIMS4,STK35,F3,OLIG1,TMEM62,PSMC1,CROP,COL20A1,ANKRD17*
**GCM-147**	9,87E-01	86	*POMGNT1,CTSE,ABHD7,SNRPD3,SLC44A2,GREB1,LOC645752,GOLPH4,SLC7A4,C5,ARNT,TBC1D4,OR5H14,PPFIA1,VPS45A,NOTCH2,MDM2,ARHGAP18,RAP1B,POLL,TNFSF8,C6orf85,LACTB2,ASB4,SRMS,NPFF,SCCPDH,PDZD4,TA-PP2C,BMPR1B,KBTBD11,TLL2,NAG,SEMA6C,MAP3K2,THAP3,WDSOF1,PBEF1,LAPTM4B,FAM19A2,PSCD3,NUP133,ZNF272,MAP2K5,WTAP,PSMD7,VIP,MECR,CD200R2,CASP12,OR5AC2,GALNT6,C9orf39,OR9K2,ZNF444,C6orf96,HECTD3,YY2,OR4M1,TNFRSF25,MRPL32,KIAA1377,BTBD5,PPP1R9B,C14orf126,PDK2,USP21,RALGDS,MRPS30,C21orf62,U18C,PLEKHB2,UBTF,CBX3,ENPP3,DDX21,ZNF193,COG4,MRPL35,ALS2,ZPBP,MRPS27,RBM19,RAB40C,HRB,TSCOT*
**GCM-148**	9,89E-01	75	*GPR25,FBN3,C1orf57,GPX1,YBX1,MYL5,ITGB3BP,CCDC12,XRCC6,C7orf31,CCDC54,GNAZ,PIGS,C20orf27,C17orf65,CAMK2B,LOC642852,BEXL1,ESCO1,IL17RE,OR4C45,WDR37,MTCH2,C6orf57,F11R,GPR110,ANKRD19,PTPRF,IMPA2,CDH18,U1SNRNPBP,DOC2A,PSKH1,LMAN1L,C9orf75,PDXK,TBC1D2B,SMAD1,COX8A,LIG4,MEP1A,CNGB3,ZNF226,INSL6,FOXR2,C15orf40,VTI1B,C12orf62,NMB,PISD,H2AFJ,ICAM4,WFDC8,CAB39,PCDHGA11,KLC2,IVD,TAS2R46,RPL23,TAF10,OR5AR1,OR6B1,ZNF336,GPR161,ZW10,SLIT2,OR4K15,NDUFS7,APC2,FRK,LGALS4,TSC1,LCE3D,FLJ20021,LSM6*
**GCM-149**	9,90E-01	24	*LOC200726,VCX,KRT12,SHBG,OR6K3,RTN3,CASP4,ZNF354A,ALPL,KRTAP10-12,FOXL1,NOPE,KRTAP19-,U101,THRB,PAGE2,PSG6,CST1,SIVA,FMN2,OR7C2,OTOP1,FGF14,SGIP1*

Abbreviations: GCM, gene coexpression module.

1.3 Table-S3. Gene overlap between the 149 GCMs and genetic findings from previous studies

GCM	GWAS RISK LOCI FOR RA	GENES TRANSCRIPTOMICALLY ASSOCIATED WITH ANTI-TNF RESPONSE IN THE RA SYNOVIUM	GENES ASSOCIATED WITH ANTI-TNF RESPONSE AT THE GENETIC LEVEL
**GCM-1**			
**GCM-2**	*SMG7*		
**GCM-3**		*PCSK7, RAPGEF1*	
**GCM-4**		*VEPH1*	
**GCM-5**		*ADRA2C, TCF4, SHC1*	
**GCM-6**			
**GCM-7**			
**GCM-8**	*RSBN1*	*PRICKLE1, SYAP1, PGD, CD163*	
**GCM-9**			
**GCM-10**		*ZNF425*	
**GCM-11**		*GSTT1, RANBP17*	
**GCM-12**		*NT5C3*	
**GCM-13**			
**GCM-14**			
**GCM-15**		*INSIG1, PLEKHA2, MFAP5, DPYSL4, STXBP6, SLK*	
**GCM-16**		*EIF4E2*	
**GCM-17**	*HLA-DRA, TAGAP, SYNGR1, MECP2*	*HLA-DPA1, RASSF4, GNRH1, GSTA4, IL6, OR6C74*	
**GCM-18**		*SYNJ2*	
**GCM-19**		*TPP1, MET, BIRC5, NEXN*	
**GCM-20**		*IL13RA2*	
**GCM-21**			
**GCM-22**		*MASP2, CTSG, ELOVL4*	
**GCM-23**			
**GCM-24**		*MYO1B, GBP7*	
**GCM-25**		*LRP2*	
**GCM-26**		*TMOD2*	
**GCM-27**			
**GCM-28**		*CXCR4*	
**GCM-29**	*TNFRSF14, PRKCH*	*USP53, C8B, TNFSF10, FANCD2, STAT2, CLIC3*	
**GCM-30**	*STAG1*		
**GCM-31**		*IL21R*	
**GCM-32**	*SPSB1*		
**GCM-33**			

**GCM-34**		*TAS2R50*	
**GCM-35**			
**GCM-36**	*AFF3, PLCL2*		
**GCM-37**		*AGPAT4*	
**GCM-38**	*NEUROD2*		
**GCM-39**			
**GCM-40**			
**GCM-41**			
**GCM-42**			
**GCM-43**			
**GCM-44**			
**GCM-45**		*RSPO3*	*EYA4*
**GCM-46**		*DLC1, HOP*	
**GCM-47**			
**GCM-48**			
**GCM-49**		*NONO*	
**GCM-50**			
**GCM-51**	*SUOX*	*ITGAX, MMP9*	
**GCM-52**	*CD200R1*	*AQP9*	
**GCM-53**		*ALDH1A3, SCARA3*	
**GCM-54**			
**GCM-55**			
**GCM-56**	*GPC5*	*RNF11*	
**GCM-57**	*RUNX1*	*FOXC2, ADAMTS5*	
**GCM-58**		*MBP*	
**GCM-59**	*EXOC2*	*TYROBP, MRPL33, ATP5H*	
**GCM-60**		*MGST2, MPP7*	
**GCM-61**			
**GCM-62**			
**GCM-63**		*IGJ, GCNT2, MED12L*	
**GCM-64**		*COCH*	
**GCM-65**			
**GCM-66**		*AXIN2*	
**GCM-67**	*HLA-DRB5, UTS2*		
**GCM-68**		*TIMP1*	
**GCM-69**	*HLA-DRB1, TRAF6*		
**GCM-70**			
**GCM-71**	*FCGR2B*		
**GCM-72**	*IL2*		
**GCM-73**	*PTPRC*	*SYPL1*	
**GCM-74**	*FCRL3, CCR6, BLK*	*RABGAP1L, VANGL1, MAP4K2, DUSP1*	*MAPK37*
**GCM-75**			
**GCM-76**			
**GCM-77**		*MPST, RDX, SLC6A8, MNDA,*	

		CDKN3, KIAA1274	
**GCM-78**			
**GCM-79**		*UBA52, SOX5, FN1, PCGF5*	
**GCM-80**		*HSPC023*	
**GCM-81**			
**GCM-82**		*SMC4L1*	
**GCM-83**			
**GCM-84**			
**GCM-85**	*SPRED2*		
**GCM-86**		*GNG4*	
**GCM-87**		*THAP11, MGLL*	
**GCM-88**			
**GCM-89**		*TFPI*	
**GCM-90**		*ZAP70*	
**GCM-91**			
**GCM-92**			
**GCM-93**			
**GCM-94**	*PLD4*	*ENPP4*	*OR10A3*
**GCM-95**		*GLDN, CDC42EP3, CXCL3*	
**GCM-96**			
**GCM-97**			
**GCM-98**			*PKD1L3*
**GCM-99**		*CORO2A*	
**GCM-100**			
**GCM-101**		*HLA-DMB*	
**GCM-102**			
**GCM-103**	*TNIP1, CD40, APOM*	*LGALS1, SCNN1B, TWISTNB, TBPL1, CHST2, DCBLD1, CDCA1, KIF23, ARL6, YPEL5*	
**GCM-104**	*CD226*		
**GCM-105**	*CASP8*	*SLAMF8, ZIC1, C7, PTGDS*	
**GCM-106**			
**GCM-107**			
**GCM-108**			
**GCM-109**		*NBN*	*ZNF595*
**GCM-110**		*RELB*	
**GCM-111**		*MCM7*	
**GCM-112**			
**GCM-113**		*ZMYND11*	
**GCM-114**	*ILF3*		
**GCM-115**	*PPIL4*	*POSTN, KCNJ13*	
**GCM-116**	*TAP2*	*TAP2*	
**GCM-117**			
**GCM-118**		*MIB2*	
**GCM-119**			

**GCM-120**	*COG6, NAV2*		*NAV2*
**GCM-121**		*SEZ6L2*	
**GCM-122**			
**GCM-123**		*TGFB2*	
**GCM-124**			
**GCM-125**			
**GCM-126**			
**GCM-127**			
**GCM-128**			
**GCM-129**	*ARL14*		
**GCM-130**		*GRPEL1*	
**GCM-131**		*FOXP1*	
**GCM-132**		*GMPR*	
**GCM-133**			
**GCM-134**	*PADI4*		
**GCM-135**	*TNFRSF9, DNASE1L3*	*GPD2*	
**GCM-136**	*UBE2L3, PADI2*	*ATP6V0A2, CATSPER3, FSCN1*	
**GCM-137**			
**GCM-138**			
**GCM-139**		*LTB*	
**GCM-140**		*PPP2R2B*	
**GCM-141**			
**GCM-142**	*ALS2CR12*	*TLE1, PDLIM5*	*CD84*
**GCM-143**		*FBP1*	
**GCM-144**	*SCT, ZPBP2*	*PCSK6, STMN1*	
**GCM-145**	*ICOSLG*		
**GCM-146**	*PTPN2*		
**GCM-147**	*C5*	*C5*	
**GCM-148**		*C15orf40*	*CDH18*
**GCM-149**			*ALPL*

Abbreviations: GCM, gene coexpression module.

1.4 Table-S4. Gene coexpression module associations with the clinical response to anti-TNF treatment at the genetic level

GCM[1]	ANTI-TNF *(P)*	ADL *(P)*	IFX *(P)*	ETN *(P)*
**GCM-1**	1,67E-01	9,93E-01	9,16E-01	8,26E-02
**GCM-2**	6,82E-01	9,40E-01	7,09E-01	4,60E-01
**GCM-3**	3,21E-01	2,13E-01	6,52E-01	8,81E-01
**GCM-4**	8,31E-01	2,05E-01	2,96E-01	4,55E-01
**GCM-5**	8,18E-01	4,51E-01	8,12E-01	6,15E-01
**GCM-6**	5,90E-01	5,25E-01	9,79E-01	2,05E-01
**GCM-7***	9,59E-01	1,60E-02	1,79E-01	1,06E-01
**GCM-8**	8,33E-01	5,87E-01	8,13E-01	1,33E-01
**GCM-9**	7,92E-01	9,26E-01	3,06E-01	9,76E-01
**GCM-10***	5,18E-01	4,28E-01	2,12E-02	6,41E-01
**GCM-11**	9,90E-01	8,28E-01	8,99E-01	8,46E-01
**GCM-12**	8,94E-01	2,56E-01	5,81E-01	6,15E-01
**GCM-13**	5,54E-01	6,60E-01	8,30E-01	8,25E-01

Abbreviations: GCM, gene coexpression module; TNF, tumor necrosis factor; ADL, clinical response to adalimumab; IFX, clinical response to infliximab; ETN, clinical response to etanercept; P, p-value.

[1] Only the 13 gene coexpression modules that were significantly associated at the transcriptomic level were tested at the genetic level.

* Gene coexpression module showing a significant association with the clinical response to the indicated treatment

1.5 Table-S5. Genes mapping to the biological processes that are nominally enriched in genes from the adalimumab-associated module

BIOLOGICAL PATHWAY	DATABASE	GENES (N)	GENES
**Metabolism of nucleotides**	Reactome	70	*ADA,PAICS,ADSSL1,ADK,CTPS1,UPP2,ADSL,ADSS,DCK,DCTD,DGUOK,DHODH,DPYD,DPYS,DTYMK,DUT,TYMP,AK1,AK2,NT5C2,GART,AK5,AMPD1,AMPD2,AMPD3,GLRX,GMPR,GPX1,GSR,GUK1,NT5C,HPRT1,APRT,IMPDH1,IMPDH2,ATIC,NME1,NME2,NME4,PNP,NT5E,RRM2B,NT5C3A,GMPR2,CMPK1,UPB1,PFAS,PPAT,CTPS2,NT5M,RRM1,RRM2,AGXT2,TK1,TK2,TXN,TXNRD1,TYMS,UCK2,UMPS,UPP1,XDH,CAD,UCK1,NT5C1A,CAT,GMPS,NT5C1B,GDA,CDA*
**GP1b-IX-V activation signaling**	Reactome	10	*FLNA,GP1BA,GP1BB,GP5,GP9,PIK3R1,RAF1,SRC,VWF,YWHAZ*
**Interleukin-7 signaling**	Reactome	11	*HGF,IL2RG,IL7,IL7R,JAK1,JAK3,PIK3R1,PIK3R2,STAT5A,STAT5B,PIK3R3"*
**Pyrimidine catabolism**	Reactome	12	*UPP2,DPYD,DPYS,TYMP,NT5C,NT5E,NT5C3A,UPB1,NT5M,AGXT2,UPP1,NT5C1A"*
**Regulation of signaling by CBL**	Reactome	18	*CRK,CRKL,FYN,GRB2,RAPGEF1,BLNK,HCK,LYN,PIK3CA,PIK3CB,PIK3CD,PIK3R1,PIK3R2,SYK,VAV1,YES1,PIK3R3,CBL*
**Synthesis and interconversion of nucleotide di- and triphosphates**	Reactome	18	*CTPS1,DTYMK,AK1,AK2,AK5,GLRX,GSR,GUK1,NME1,NME2,NME4,RRM2B,CMPK1,CTPS2,RRM1,RRM2,TXN,TXNRD1*
**Tie2 Signaling**	Reactome	18	*ANGPT1,ANGPT2,GRB2,GRB7,GRB14,HRAS,KRAS,NRAS,ANGPT4,PIK3CA,PIK3CB,PIK3R1,PIK3R2,PTPN11,SHC1,SOS1,TEK,DOK2*
**Signaling by constitutively active EGFR**	Reactome	19	*CDC37,EGF,EGFR,GAB1,GRB2,HRAS,HSP90AA1,KRAS,NRAS,PIK3CA,PIK3R1,PLCG1,RPS27A,SHC1,SOS1,UBA52,UBB,UBC,CBL*
**Nephrin interactions**	Reactome	22	*CD2AP,FYN,NCK1,NPHS1,PIK3CA,PIK3CB,PIK3R1,PIK3R2,KIRREL1,SPTAN1,SPTBN1,ACTN4,KIRREL2,NCK2,KIRREL3,CASK,ACTN1,ACTN2,IQGAP1,ACTN3,WASL,MAGI2*
**Pyrimidine metabolism**	Reactome	24	*UPP2,DCK,DCTD,DHODH,DPYD,DPYS,DUT,TYMP,NT5C,NT5E,NT5C3A,UPB1,NT5M,AGXT2,TK1,TK2,TYMS,UCK2,UMPS,UPP1,CAD,UCK1,NT5C1A,CDA*
**Interleukin receptor SHC signaling**	Reactome	28	*CSF2,CSF2RA,CSF2RB,GRB2,HRAS,IL2,IL2RA,IL2RB,IL2RG,IL3,IL3RA,IL5,IL5RA,INPP5D,INPPL1,JAK1,JAK2,JAK3,PIK3CA,PIK3CB,PIK3CD,PIK3R1,PIK3R2,PTPN6,SHC1,SOS1,PIK3R3,GAB2*
**CD28 co-stimulation**	Reactome	31	*AKT3,THEM4,MAP3K8,AKT1,AKT2,MTOR,RICTOR,FYN,GRB2,LCK,LYN,PAK1,PAK2,PDPK1,PIK3CA,PIK3R1,PIK3R2,TRIB3,RAC1,MLST8,SRC,VAV1,YES1,MAPKAP1,PIK3R3,MAP3K14,CD28,GRAP2,CD80,CD86,CDC42*
**GPVI-mediated activation cascade**	Reactome	33	*AKT3,VAV3,COL1A1,COL1A2,PIK3R6,AKT1,AKT2,FCER1G,PIK3R5,FYN,LAT,RHOA,RHOB,RHOG,LCK,LCP2,LYN,GP6,PDPK1,PIK3CA,PIK3CB,PIK3CG,PIK3R1,PIK3R2,PLCG2,PRKCZ,RAC1,RAC2,SYK,VAV1,VAV2,PIK3R3,CDC42*

**PI3K/AKT activation**	Reactome	37	*AKT3,CDKN1A,CDKN1B,CHUK,THEM4,CREB1,AKT1,AKT2,FOXO1,FOXO3,PHLPP1,MTOR,RICTOR,GSK3A,GSK3B,NR4A1,IRS1,RHOA,MDM2,FOXO4,NGF,NTRK1,PDPK1,PIK3CA,PIK3CB,PIK3R1,PIK3R2,BAD,PTEN,TRIB3,RPS6KB2,MLST8,TSC2,MAPKAP1,CASP9,AKT1S1,IRS2*
**PI3K events in ERBB4 signaling**	Reactome	38	*AKT3,CDKN1A,CDKN1B,NRG3,CHUK,THEM4,CREB1,NRG4,HBEGF,ERBB4,EREG,AKT1,AKT2,FOXO1,FOXO3,PHLPP1,MTOR,RICTOR,GSK3A,GSK3B,NRG1,NR4A1,MDM2,FOXO4,PDPK1,PIK3CA,PIK3R1,BAD,PTEN,TRIB3,RPS6KB2,MLST8,BTC,TSC2,MAPKAP1,CASP9,AKT1S1,NRG2*
**GAB1 signalosome**	Reactome	39	*AKT3,CDKN1A,CDKN1B,CHUK,THEM4,CREB1,CSK,EGF,EGFR,AKT1,AKT2,FOXO1,FOXO3,PHLPP1,MTOR,RICTOR,GAB1,GRB2,GSK3A,GSK3B,NR4A1,MDM2,FOXO4,PDPK1,PIK3CA,PIK3R1,PAG1,BAD,PTEN,TRIB3,PTPN11,PXN,RPS6KB2,MLST8,SRC,TSC2,MAPKAP1,CASP9,AKT1S1*
**Interleukin-2 signaling**	Reactome	42	*CSF2,CSF2RA,CSF2RB,PTK2B,GRB2,HRAS,IL2,IL2RA,IL2RB,IL2RG,IL3,IL3RA,IL5,IL5RA,INPP5D,INPPL1,JAK1,JAK2,JAK3,KRAS,LCK,NRAS,PIK3CA,PIK3CB,PIK3CD,PIK3R1,PIK3R2,MAPK1,MAPK3,MAP2K1,MAP2K2,PTPN6,RAF1,SHC1,SOS1,STAT5A,STAT5B,SYK,YWHAB,PIK3R3,CDK1,GAB2*
**PI3K events in ERBB2 signaling**	Reactome	44	*AKT3,CDKN1A,CDKN1B,NRG3,CHUK,THEM4,CREB1,NRG4,HBEGF,EGF,EGFR,ERBB2,ERBB3,ERBB4,EREG,AKT1,AKT2,FOXO1,FOXO3,PHLPP1,MTOR,RICTOR,GAB1,GRB2,GSK3A,GSK3B,NRG1,NR4A1,MDM2,FOXO4,PDPK1,PIK3CA,PIK3R1,BAD,PTEN,TRIB3,RPS6KB2,MLST8,BTC,TSC2,MAPKAP1,CASP9,AKT1S1,NRG2*
**Interleukin-3, 5 and GM-CSF signaling**	Reactome	46	*TEC,CRK,CRKL,CSF2,CSF2RA,CSF2RB,FYN,GRB2,RAPGEF1,BLNK,HCK,HRAS,IL2,IL2RA,IL2RB,IL2RG,IL3,IL3RA,IL5,IL5RA,INPP5D,INPPL1,JAK1,JAK2,JAK3,LYN,PIK3CA,PIK3CB,PIK3CD,PIK3R1,PIK3R2,PRKACB,PTPN6,PTPN11,SHC1,SOS1,STAT5A,STAT5B,SYK,TEC,VAV1,YES1,YWHAZ,PIK3R3,CBL,GAB2*
**Downstream TCR signaling**	Reactome	50	*MALT1,CHUK,TAB2,TRBV12-3,TRBC1,TRAV19,TRAV8-4,TRAC,HLA-DMA,HLA-DMB,HLA-DOA,HLA-DOB,HLA-DPA1,HLA-DPB1,HLA-DQA1,HLA-DQA2,HLA-DQB1,HLA-DQB2,HLA-DRA,HLA-DRB1,HLA-DRB3,HLA-DRB4,HLA-DRB5,IKBKB,INPP5D,LCK,NFKB1,NFKBIA,TRAT1,PDPK1,PIK3CA,PIK3CB,PIK3R1,PIK3R2,PRKCQ,PTEN,RELA,MAP3K7,TRAF6,UBE2N,UBE2V1,CARD11,IKBKG,RIPK2,BCL10,CD3D,CD3E,CD3G,CD247,CD4*
**Mitochondrial Protein Import**	Reactome	52	*TOMM6,TIMM23,COX17,SLC25A13,TIMM17B,TIMM17A,TOMM40,TIMM44,MTX2,DNAJC19,CHCHD4,GRPEL2,CS,HSCB,CYC1,TIMM8A,LDHD,PMPCA,FXN,SAMM50,TIMM10B,TIMM13,TIMM10,TIMM9,TIMM8B,GFER,COQ2,TIMM21,SLC25A4,SLC25A6,TIMM22,HSPA9,HSPD1,IDH3G,TOMM5,MTX1,ATP5F1A,ACO2,ATP5F1B,PAM16,ATP5MC1,TOMM7,TOMM22,BCS1L,TAZ,VDAC1,GRPEL1,SLC25A12,TIMM50,PMPCB,TOMM20,TOMM70*

**Antigen Activates B Cell Receptor Leading to Generation of Second Messengers**	Reactome	53	*PIK3AP1,FYN,DAPP1,IGKV1-5,IGHV7-81,IGHV3-23,IGHV1-46,IGLV7-43,IGLV1-51,IGLC7,GRB2,IGKV1D-16,IGKV5-2,IGKV4-1,IGKV3-20,IGKV3-15,IGKV3-11,IGKV3-7,IGKV2-30,IGKV1-39,BLNK,SH3KBP1,IGHD,IGHM,IGKC,IGLC1,IGLC2,IGLC3,IGLC6,ITPR1,ITPR2,ITPR3,LYN,NCK1,PIK3CD,PIK3R1,PLCG1,PLCG2,BLK,SHC1,SOS1,STIM1,SYK,BTK,TRPC1,VAV1,CALM1,ORAI1,CBL,CBLB,CD19,CD79A,CD79B*
**PI-3K cascade**	Reactome	57	*AKT3,CDKN1A,CDKN1B,FRS2,CHUK,THEM4,CREB1,KLB,AKT1,AKT2,FGF1,FGF2,FGF3,FGF4,FGF5,FGF6,FGF7,FGF8,FGF9,FGF10,FGFR1,FGFR3,FGFR2,FGFR4,FOXO1,FOXO3,PHLPP1,MTOR,RICTOR,GAB1,FGF20,FGF22,GRB2,GSK3A,GSK3B,NR4A1,MDM2,FOXO4,PDPK1,PIK3CA,PIK3R1,BAD,PTEN,TRIB3,PTPN11,RPS6KB2,MLST8,TSC2,MAPKAP1,FGF23,CASP9,AKT1S1,FGF18,FGF17,FGF16,KL,FGF19*
**TCR signaling**	Reactome	67	*MALT1,CHUK,CSK,TAB2,FYB1,LAT,TRBV12-3,TRBC1,TRAV19,TRAV8-4,TRAC,HLA-DMA,HLA-DMB,HLA-DOA,HLA-DOB,HLA-DPA1,HLA-DPB1,HLA-DQA1,HLA-DQA2,HLA-DQB1,HLA-DQB2,HLA-DRA,HLA-DRB1,HLA-DRB3,HLA-DRB4,HLA-DRB5,IKBKB,INPP5D,ITK,LCK,LCP2,NCK1,NFKB1,NFKBIA,PAK1,PAK2,TRAT1,EVL,PDPK1,PIK3CA,PIK3CB,PIK3R1,PIK3R2,PLCG1,ENAH,PAG1,PRKCQ,PTEN,PTPRC,RELA,MAP3K7,TRAF6,UBE2N,UBE2V1,VASP,WAS,ZAP70,CARD11,IKBKG,RIPK2,BCL10,CD3D,CD3E,CD3G,CD247,CD4,GRAP2*
**PI3K Cascade**	Reactome	70	*FRS2,THEM4,KLB,DOK1,EIF4B,EIF4E,EIF4EBP1,EIF4G1,AKT2,FGF1,FGF2,FGF3,FGF4,FGF5,FGF6,FGF7,FGF8,FGF9,FGF10,FGFR1,FGFR3,FGFR2,FGFR4,MTOR,GAB1,FGF20,FGF22,GRB2,EEF2K,PIK3R4,INS,INSR,IRS1,PDE3B,PRKAG2,PDPK1,CAB39,PIK3C3,PIK3CA,PIK3CB,PIK3R1,PIK3R2,PRKAG3,TLR9,PPM1A,STRADB,PRKAA1,PRKAA2,PRKAB1,PRKAB2,PRKAG1,RPTOR,TRIB3,PTPN11,RHEB,RPS6,RPS6KB1,MLST8,STK11,TSC1,TSC2,FGF23,CAB39L,IRS2,FGF18,FGF17,FGF16,STRADA,KL,FGF19*
**Costimulation by the CD28 family**	Reactome	76	*AKT3,THEM4,MAP3K8,CSK,CTLA4,BTLA,AKT1,AKT2,ICOSLG,MTOR,RICTOR,FYN,TRBV12-3,TRBC1,TRAV19,TRAV8-4,TRAC,GRB2,CD274,ICOS,HLA-DMA,HLA-DMB,HLA-DOA,HLA-DOB,HLA-DPA1,HLA-DPB1,HLA-DQA1,HLA-DQA2,HLA-DQB1,HLA-DQB2,HLA-DRA,HLA-DRB1,HLA-DRB3,HLA-DRB4,HLA-DRB5,LCK,LYN,PAK1,PAK2,PDCD1,PDPK1,PIK3CA,PIK3R1,PIK3R2,PPP2CA,PPP2CB,PPP2R1A,PPP2R1B,PPP2R5A,PPP2R5B,PPP2R5C,PPP2R5D,PPP2R5E,TRIB3,PTPN6,PTPN11,RAC1,MLST8,SRC,VAV1,YES1,MAPKAP1,PDCD1LG2,PIK3R3,TNFRSF14,MAP3K14,CD3D,CD3E,CD3G,CD247,CD4,CD28,GRAP2,CD80,CD86,CDC42*
**G alpha (12/13) signalling events**	Reactome	77	*NET1,VAV3,GNA13,GNB5,AKAP13,FGD4,ADRA1D,ADRA1B,ADRA1A,ECT2,FGD2,FGD1,ARHGEF9,MCF2L,ARHGEF12,ARHGE*

			F18,NGEF,TIAM2,ARHGEF16,GNA12,GNB1,GNB2,GNB3,GNG3,GNG4,GNG5,GNG7,GNG10,GNG11,GNGT1,GNGT2,ABR,RHOA,RHOB,RHOC,RHOG,ARHGAP4,MCF2,ARHGEF4,ARHGEF3,GNG13,PIK3CA,PIK3R1,PIK3R2,PLXNB1,GNG2,GNG12,PLEKHG5,PREX1,RAC1,RAC2,RASGRF1,RASGRF2,GNB4,ROCK1,ITSN1,PLEKHG2,SOS1,SOS2,TBXA2R,TIAM1,TRIO,VAV1,VAV2,OBSCN,PIK3R3,ARHGEF7,FGD3,KALRN,ARHGEF1,ARHGEF2,GNG8,ARHGEF6,ROCK2,ARHGEF11,ARHGEF17,CDC42
**Signaling by SCF-KIT**	Reactome	79	*AKT3,TEC,SH2B3,PTPRU,CDKN1A,CDKN1B,SH2B2,GRAP,CHEK1,CHUK,THEM4,CMA1,CREB1,AKT1,AKT2,FER,FES,FOXO1,FOXO3,PHLPP1,MTOR,RICTOR,FYN,GRB2,GRB7,GRB10,GSK3A,GSK3B,NR4A1,HRAS,JAK2,KIT,KRAS,LCK,LYN,MDM2,KITLG,FOXO4,MMP9,NRAS,PDPK1,PIK3CA,PIK3R1,PIK3R2,PRKCA,MAPK1,MAPK3,MAP2K1,MAP2K2,BAD,PTEN,TRIB3,PTPN6,PTPN11,RAC1,RAF1,RPS6KB2,MLST8,SOS1,SRC,STAT1,STAT3,STAT5A,STAT5B,TEC,TSC2,VAV1,YES1,YWHAB,MAPKAP1,CASP9,AKT1S1,PIK3R3,SOCS1,CBL,SOCS6,GRAP2,CDK1,GAB2*
**IRS-mediated signalling**	Reactome	81	*FRS2,THEM4,KLB,DOK1,EIF4B,EIF4E,EIF4EBP1,EIF4G1,AKT2,FGF1,FGF2,FGF3,FGF4,FGF5,FGF6,FGF7,FGF8,FGF9,FGF10,FGFR1,FGFR3,FGFR2,FGFR4,MTOR,GAB1,FGF20,FGF22,GRB2,EEF2K,PIK3R4,HRAS,INS,INSR,IRS1,KRAS,NRAS,PDE3B,PRKAG2,PDPK1,CAB39,PIK3C3,PIK3CA,PIK3CB,PIK3R1,PIK3R2,PRKAG3,TLR9,PPM1A,STRADB,PRKAA1,PRKAA2,PRKAB1,PRKAB2,PRKAG1,MAPK1,MAPK3,MAP2K1,MAP2K2,RPTOR,TRIB3,PTPN11,RAF1,RHEB,RPS6,RPS6KB1,MLST8,SOS1,STK11,TSC1,TSC2,YWHAB,FGF23,CAB39L,IRS2,FGF18,FGF17,FGF16,STRADA,KL,CDK1,FGF19*
**IRS-related events**	Reactome	81	*FGF9,FGF10,EIF4B,EIF4E,EIF4EBP1,FGFR1,FGFR3,FGFR2,FGFR4,MTOR,GAB1,FGF20,FGF22,GRB2,FRS2,THEM4,KLB,DOK1,EIF4G1,IRS1,KRAS,NRAS,PDE3B,PRKAG2,PDPK1,CAB39,PIK3C3,PIK3CA,PIK3CB,PIK3R1,PIK3R2,PRKAG3,TLR9,PPM1A,STRADB,PRKAA1,PRKAA2,AKT2,FGF1,FGF2,FGF3,FGF4,FGF5,FGF6,FGF7,FGF8,EEF2K,PIK3R4,HRAS,INS,INSR,PRKAB1,PRKAB2,PRKAG1,FGF17,FGF16,STRADA,KL,CDK1,FGF19,MAP2K2,RPTOR,MAPK1,MAPK3,MAP2K1,TRIB3,PTPN11,RAF1,RHEB,RPS6,RPS6KB1,MLST8,SOS1,STK11,TSC1,TSC2,YWHAB,FGF23,CAB39L,IRS2,FGF18*

2. Supplementary Figures

2.1 Figure S1. Principal components of the RA cohort used in the discovery stage of the genetic association analysis

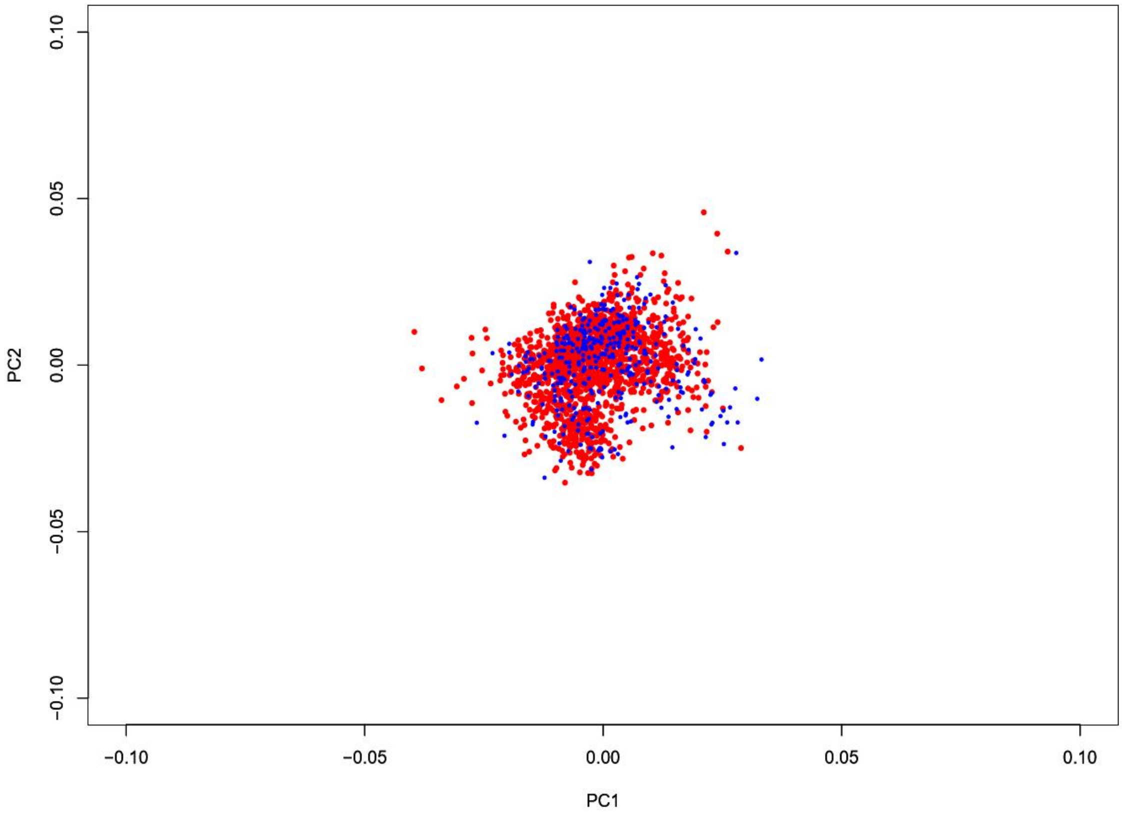

The anti-TNF treated rheumatoid arthritis patients (blue dots) and control patients (red dots) are plotted according to their first (PC1) and second (PC2) principal components estimated in EIGENSTRAT using the GWAS data.

2.2 Figure S2. Nucleotide metabolism pathway integrating GCM and SNP information

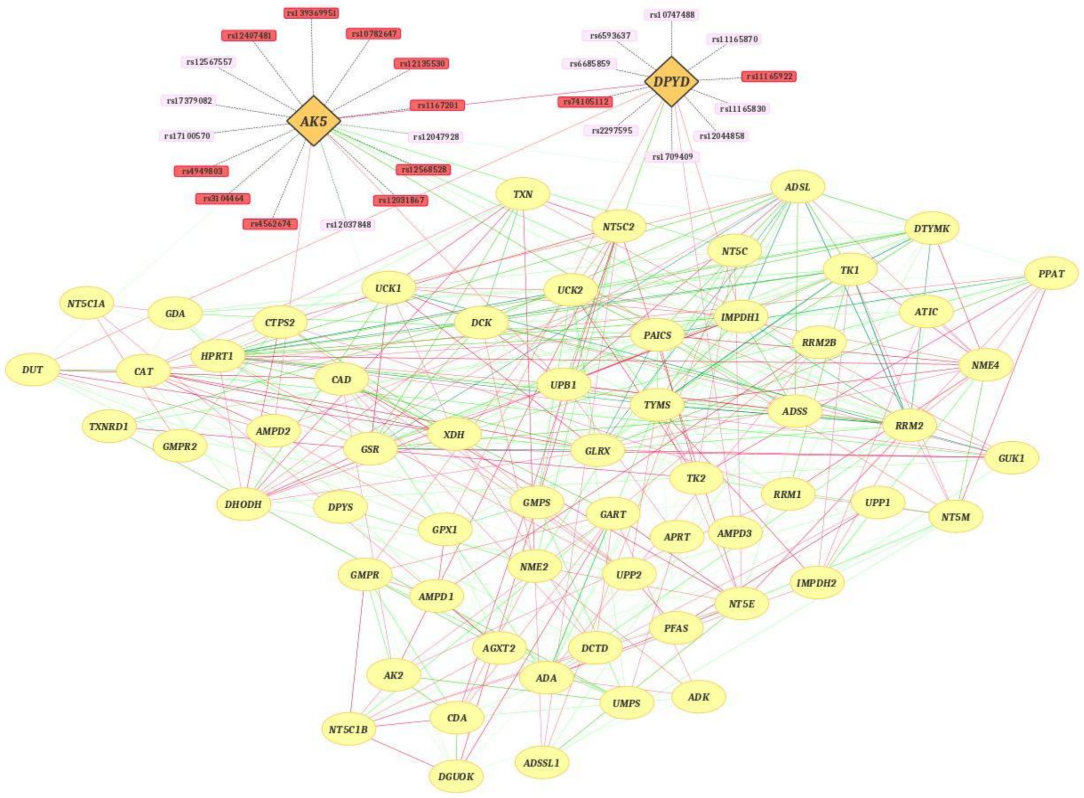

Gene co-expression network illustrating the nucleotide metabolism pathway in the RA synovium. In this network, genes are connected according to their gene expression correlation in the synovial tissue. Green and red lines indicate positive and negative correlation, respectively. The *AK5* and *DPYD* genes from the adalimumab-associated module (i.e. GCM-7) are represented as orange diamonds. The SNPs from the GCM-7 that were found to be associated with adalimumab response (P<0.05) are connected to their assigned genes by a dashed black line. The associated SNPs that are not in linkage disequilibrium in the discovery and replication cohorts (r^2<0.2) are colored in red and pink, respectively.

2.3 Figure S3. Epigenetic fine-mapping of genetic variation from the GCM-7 and the cell-type specific H3K27ac and H3K4me1 marks.

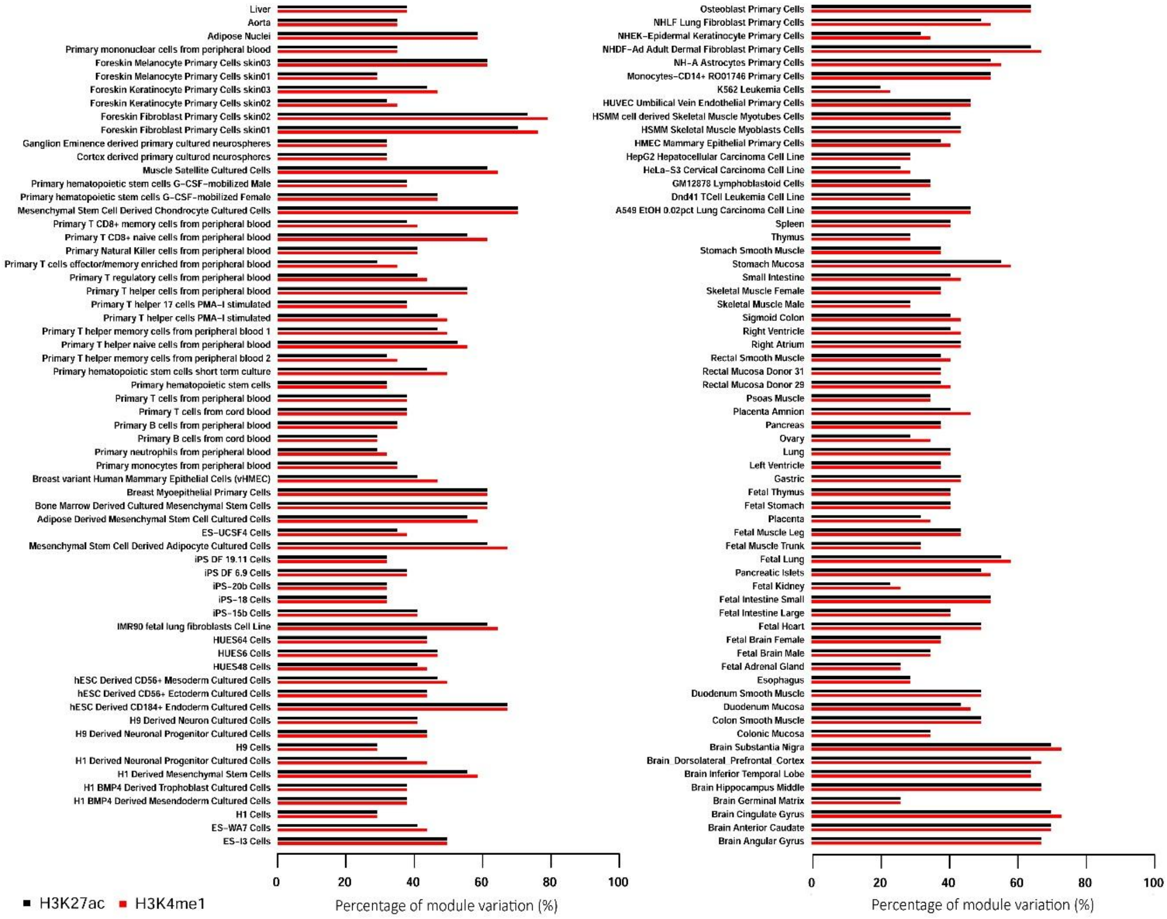

Percentage of genetic variation from the GCM-7 mapping on cell-type specific enhancer histone marks. Following the same approach that we used to perform the cell-type epigenetic enrichment analysis on H3K4me3, the present epigenetic fine-mapping was focused on adalimumab-associated variants from the GCM-7 (P<0.05 in either the discovery or replication cohorts) that are not in linkage disequilibrium (r^2<0.2). The cell-type specific epigenetic histone marks H3K27ac and H3K4me1 are colored in black and red, respectively.
